# Synthesis of a Di-Mycoloyl Tri-Arabinofuranosyl Glycerol Fragment of the Mycobacterial Cell Wall, Based on Synthetic Mycolic Acids

**DOI:** 10.3390/molecules24193596

**Published:** 2019-10-06

**Authors:** Omar T. Ali, Mohsin O. Mohammed, Juma’a R. Al Dulayymi, Mark S. Baird

**Affiliations:** 1 School of Chemistry, Bangor University, Bangor, Wales LL57 2UW, UK; omarkemia@yahoo.com.hk (O.T.A.); althker1@yahoo.com (M.O.M.); 2 School of Natural Sciences, Bangor University, Bangor, Wales LL57 2UW, UK

**Keywords:** mycobacteria, cell membrane, mycolic acids, triarabinoglycerol

## Abstract

Fragments of mycobacterial cell walls such as arabinoglycerol mycolate and dimycoloyl diarabinoglycerol, comprising complex mixtures of mycolic acids, have immunostimulatory and antigenic properties. A related di-mycoloyl tri-arabinofuranosyl glycerol fragment has been isolated from cell wall hydrolysates. An effective stereoselective synthesis of tri-arabinofuranosyl glycerol, followed by coupling with stereochemically defined mycolic acids of different structural classes, to provide unique di-mycoloyl tri-arabinofuranosyl glycerols is now described.

## 1. Introduction

**Figure 1:**
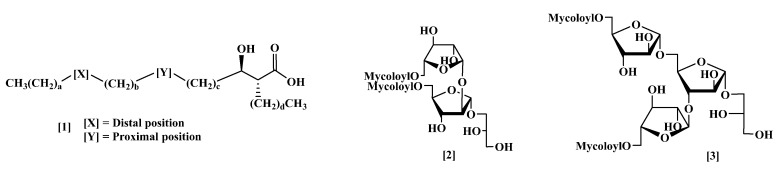
Generalised structure of mycolic acids (**1**), dimycoloyl diarabinoglycerol (DMAG) (**2**) and dimycoloyl triarabinoglycerol (DMTAG) (**3**).

The cell wall in *Mycobacterium tuberculosis* and in other mycobacteria has an unusual structure, containing a multi-layered and extremely hydrophobic envelope, which is important for the organism to survive in macrophages, and includes characteristic complex mixtures of long-chain (C70–C90), α-alkyl branched β-hydroxylated fatty acids, ‘mycolic acids’ (MA); these include a range of groups (Figure 1), including *cis-* and *trans*-cyclopropanes, and *cis*- and *trans*-alkenes at positions X or Y and methoxy and keto-groups at position X [1,2]. The mycoloyl–arabinogalactan complex (mAG), the largest component structure in the cell wall of mycobacteria, is believed to act as a permeability barrier that prevents the passage of antibiotics. As well as being bound to the wall, largely as penta-arabinose tetramycolates, MA are also present as non-wall-bound sugar esters, such as trehalose dimycolate (TDM) and monomycolate (TMM), and as free acids. Hydrolysis of the cell wall of *Mycobacterium bovis* gave a penta-arabinose tetramycolate, and an arabinose *mono*-mycolate, as well as hexa-arabinose, hepta-arabinose and octa-arabinose tetramycolates [3]. Isolated natural arabinomycolates possess potent adjuvant immunostimulatory activity [4,5]. The preparation of a tetramycoloyl penta-arabinose using a complex natural mixture of MAs has been described [6,7,8,9,10]. Such fragments have also been found to be of value in the treatment of cancer [11]. Smaller fragments, such as glycerol mycolate [12,13,14,15,16,17] and arabinoglycerol mycolate [18,19], have also been reported and, in the former case, have significant biological activity. In 1992, a new glycolipid, dimycoloyl diarabinoglycerol (DMAG) (**2**), was isolated from the *Mycobacterium avium*–*Mycobacterium intracellulare* complex (MAC) [20]. High immunoglobulin M (IgM) titres against the glycolipid were observed in enzyme-linked immunosorbent assays (ELISA) of serum from individuals who were culture positive for MAC infection, implying that this serodiagnosis detects the disease in an active phase [21]. A similar glycolipid mixture was isolated from *M. bovis* Bacille Calmette-Guérin or *Mycobacterium marinum* and from *M. tuberculosis* [22]. The DMAG from *M. marinum* was found to induce the secretion of proinflammatory cytokines (tumour necrosis factor α (TNF-α), and interleukins (IL-8, and IL-1β)) in human macrophage THP-1 cells, to trigger the expression of the protein ICAM-1 and cluster of differentiation 40 (CD40) cell surface antigens, and to modulate genes related to immune and inflammatory responses, suggesting that DMAG may drive host–pathogen interactions and participate in the immunopathogenesis of mycobacterial infections [23]. There is one report of the isolation of a dimycoloyl triarabinoglycerol (DMTAG, **3**) from the degradation of the cell mycoloylarabinogalactan–peptidoglycan–protein complex from *M. tuberculosis* [24]. 

We have already reported the synthesis of triarabinose dimycolates [25], of arabinoglycerol mycolates [19], and of DMAGs containing unique synthetic mycolic acids [26]. Although, so far, it has no reported bioactivity, we now report the extension of the methods used in those synthetic approaches to the synthesis of a single stereoisomer of tri-arabinofuranosyl glycerol (TAG) and of a series of stereo-defined DMTAG glycolipids (Figure 1), through esterification of the glycan with structurally defined synthetic MAs of α- and keto-classes [1,2]. 

## 2. Results and Discussion

**Scheme 1:**
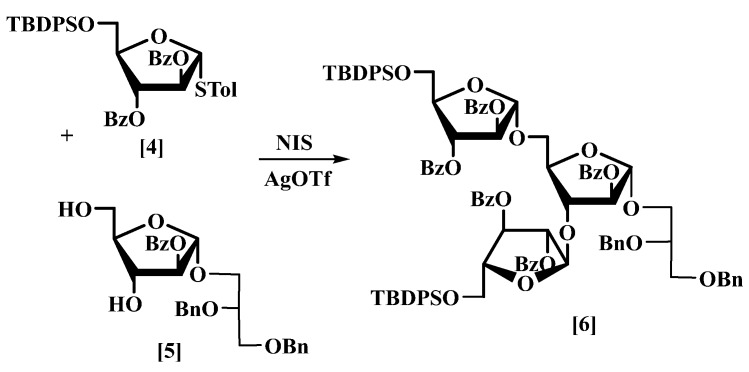
Synthesis of fully protected glycan tri-arabino glycerol (TAG) (**6**).

The target tri-saccharide structure (**6**) has three α-glycosidic linkages and can be assembled readily from a donor (**4**) [18,19] and a diol acceptor (**5**) (Scheme 1), a similar approach to the synthesis of the methoxy tri-saccharide of D-arabinofuranoside [25].

**Scheme 2:**
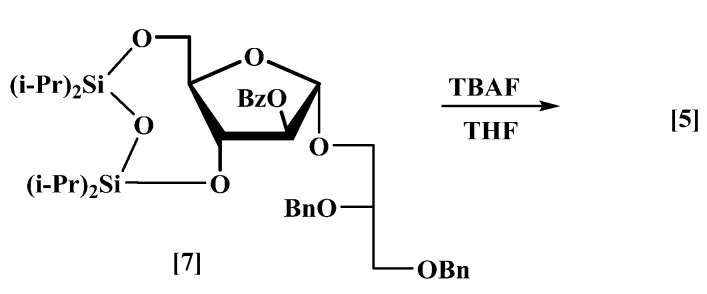
Synthesis of the acceptor (**5**).

The donor (**4**) was prepared before [18,19]. The target acceptor (**5**) was obtained in 95% yield by desilylation of the protected arabinofuranosyl glycerol (**7**), prepared from d-arabinose [20], using tetrabutylammmonium fluoride in dry THF to give two free hydroxyl groups at the C-3 and C-5 positions, respectively (Scheme 2).

Following the method adopted by Liu et al. [6], the thioglycoside donor (**4**) was coupled with the acceptor (**5**) in the presence of N-iodosuccinimide (NIS)/silver trifluromethane sulfonate (AgOTf) in dry CH_2_Cl_2_ to give (**6**) in 91% yield. The ^1^H-NMR spectrum of (**6**) included three broad singlets at δ 5.61, 5.31 and 5.22, corresponding to the protons at the anomeric centre of each ring. The ^13^C-NMR spectrum (Figure S1, supporting information) established the presence of the α-glycosidic linkages in the tri-arabinofuranosyl glycerol (**6**), with the signals at δ 106.1 and 105.2 ppm belonging to the three carbons at the anomeric centres. The HSQC-NMR of (**6**) (Figure S2), showed the three peaks corresponding to the acetal protons, at δ 5.61, 5.31 and 5.22, correlated to their carbons, in agreement with the assignments made by Liu et al. [6,25].

**Scheme 3:**
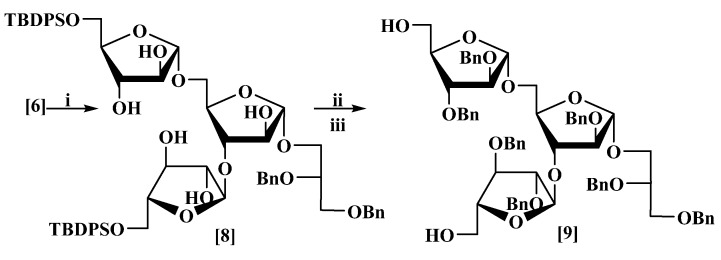
Synthesis of compound (**9**). Reagents and conditions: (i) MeONa (1 M), then ethanoic acid (AcOH), 83%; (ii) benzyl bromide (BnBr), NaH, dimethylformamide (DMF), 65%; (iii) tetrabutylammonium fluoride (TBAF), tetrahydrofuran (THF), 87%.

The tri-saccharide (**6**) was debenzoylated with sodium methoxide to give (**8**) as a thick oil in 83% yield; in the ^1^H-NMR of (**8**), all the signals corresponding to the protons on the carbon adjacent to the benzoyl ester (**6**) were shifted up-field. The ^13^C-NMR spectrum showed the disappearance of the carbonyl signals. Compound (**8**) was benzylated to protect the five secondary hydroxyl groups using benzyl bromide (BnBr) and sodium hydride in dry dimethylformamide (DMF) in 65% yield, followed by de-protection of the two primary hydroxyl groups using tetrabutylammonium fluoride (TBAF) to afford (**9**) in 87% yield (Scheme 3). 

Compound (**9**) was then esterified, either by an alkylative coupling using cesium hydrogen carbonate after mesylation of the primary hydroxyl groups in the glycan or by direct coupling with a fatty acid using 1-ethyl-3-(3-dimethylaminopropyl)carbodi-imide (EDCI). 

**Scheme 4:**
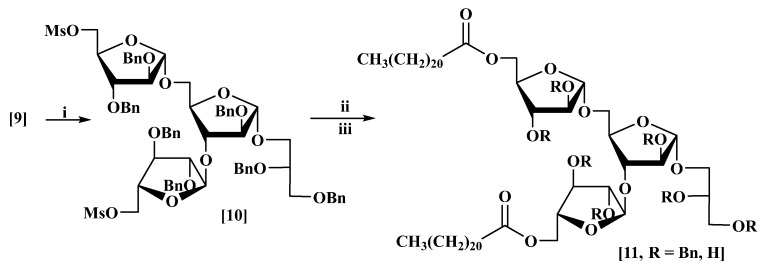
Synthesis of a model di-behenoyl tri-arabinoglycerol (**11**, R = H). Reagents and conditions: (i) methanesulfonyl chloride (MsCl), 4-dimethylaminopyridine (DMAP), pyridine, 87%; (ii) CsHCO_3_, behenic acid, DMF/THF, 70 °C, (**11**, R = Bn, 80%); (iii) Pd(OH)_2_/C (20%), H_2_, 82%.

In the first method, the two primary hydroxyl groups in (**9**) were activated using methanesulfonyl chloride (MsCl) in dry pyridine in the presence of catalytic 4-dimethylamino-pyridine (DMAP) in dry CH_2_Cl_2_ at 0 °C to afford the corresponding mesylate (**10**) in 87% yield (Scheme 4). The ^1^H-NMR spectrum of (**10**) (Figure S4) showed the expected signals, including two singlets at δ 2.94 and 2.89 for the methyl groups of the mesylates. The ^13^C-NMR spectrum (Figure S5) showed two signals at δ 37.6 and 37.5 for the carbons of the mesylates. The assignments of the signals were made by comparison with literature values reported for the methoxy tri-arabinose compound, which is identical to (**10**) except for the absence of the glycerol moiety [16,17]. 

The protected di-behenoyl-triarabinoglycerol (**11**, R = Bn) was prepared in 80% yield by coupling the mesylate (**10**) with behenic acid using cesium hydrogen carbonate in dry THF: DMF at 70 °C for 3 days (Scheme 4). 

The ^1^H-NMR spectrum showed three broad one-proton singlets at δ 5.09, 5.06 and 4.97, corresponding to the three protons at the anomeric centres on the glycan rings. The CH_2_ groups adjacent to the carbonyls gave a triplet at δ 2.17 (*J* 7.6 Hz) integrating to four protons. The terminal methyl group showed a triplet signal at δ 0.81 (*J* 6.8 Hz) integrating to six protons. The ^13^C-NMR spectrum showed two signals at δ 173.6 and 173.5 for the carbonyl groups. Signals corresponding to the carbon at the anomeric centre for the three rings appeared at δ 106.5, 106.2 and 105.5. The carbon of the CH_2_ group adjacent to the carbonyl in the acid appeared at δ 34.1. The methylene chain ranged from δ 32–22 and the terminal methyl group at δ 14.1. 

Compound (**11**, R = Bn) was debenzylated by stirring vigorously in a suspension of Pd(OH)_2_/C in dry CH_2_Cl_2_:MeOH:THF (1:1:1.5) under an atmosphere of hydrogen for 36 h to give the target DMTAG analogue (**11**, R = H) in 82% yield (Scheme 4). The ^1^H-NMR spectrum (Figure S7) of compound (**11**, R = H) showed three broad singlets at δ 5.01, 4.97 and 4.90 ppm for the three α-protons, with the remaining 20 protons of the sugar and glycerol moieties appearing between δ 4.30 and 3.50 ppm. The four protons next to the two carbonyls gave a triplet at δ 2.30 ppm (*J* 7.6 Hz), while the terminal methyl groups appeared at δ 0.83 ppm as a triplet (*J* 6.5 Hz). The ^13^C-NMR spectrum obtained for the glycolipid analogue (**11**, R = H) gave signals (Figure S8), which were essentially identical to those for an analogue in the literature [25], bearing a methoxy substituent at C-1 rather than the glycerol substituent in compound (**11**, R = H), and are assigned on that basis (See Supplementary Information, Table S1). 

**Scheme 5:**
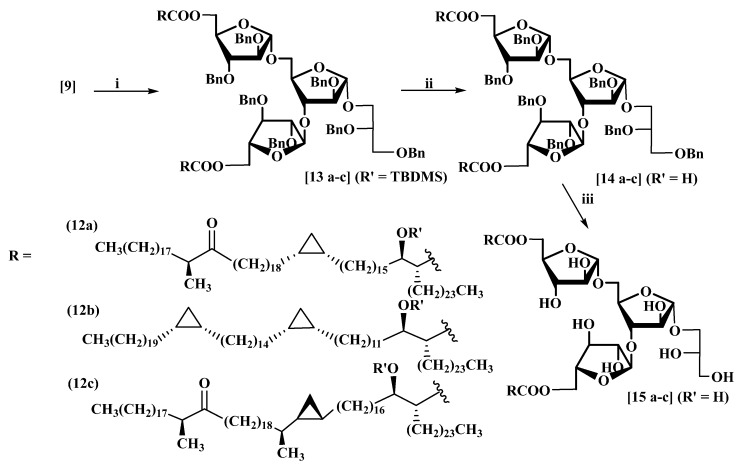
Synthesis of dimycoloyl triarabinoglycerol (DMTAG) glycolipids. (i) RCOOH (R’ = TBDMS) (**12a**–**c**), 1-ethyl-3-(3-dimethylaminopropyl)carbodi-imide (EDCI), DMAP, CH_2_Cl_2_ (a: 51%; b: 84%; c: 46%); (ii) TBAF, THF (a 76%; b: 51%; c: 66%); (iii) Pd(OH)_2_/C (20%), H_2_, CH_3_OH:CH_2_Cl_2_: THF (1:1:1.5), rt, 36 h, (a: 86%; b: 88%; c: 82%).

The effects of both free mycolic acids and of their sugar esters on immune responses, and on their recognition by disease antibodies, are known to depend on the specific classes they comprise, and on the detail of their stereochemistry [2,27,28,29]. The glycan (**9**) was therefore then coupled with three common classes of structurally defined synthetic MAs, *cis*-cyclopropane containing keto-MA (**12a**), α-MA (**12b**) and *trans*-cyclopropane containing keto-MA (**12c**) [30,31,32], followed by deprotection, as in Scheme 5, to provide examples of three classes for the evaluation of their effects in these areas. In these cases, the coupling was achieved using EDCI-DMAP, which gave somewhat better yields based on the protected synthetic mycolic acids [19,25].

The effects of the synthetic triarabinoglycerol mycolates in cytokine and chemokine stimulation, and their recognition by disease antibodies, are currently under investigation.

## 3. Methods

### 3.1. General

The chemicals used were obtained from commercial suppliers (Sigma-Aldrich, Lee on Solent, UK, and Alfa Aesar, Heysham, UK) or prepared from them by the methods described. Ether and tetrahydrofuran were dried over sodium wire and benzophenone under nitrogen, while dichloromethane was dried over calcium hydride. The petroleum spirit (petrol) used had boiling point of 40–60 °C. All reagents and solvents used were of reagent grade unless otherwise stated. Silica gel (Merck 7736) used for column and thin-layer chromatography was obtained from Sigma; separated components were detected variously using UV light, I_2_ and phosphomolybdic acid solution in IMS followed by charring. Anhydrous MgSO_4_ was used to dry organic solutions. Infrared (IR) spectra were carried out on a Perkin–Elmer 1600 F.T.I.R. spectrometer using liquid films or a KBr disc (solid). NMR spectra were carried out on Bruker Avance 400 or 500 spectrometers. [α]_D_ values were recorded in CHCl_3_ on a POLAAR 2001 optical activity polarimeter. Matrix-assisted laser desorption/ionization (MALDI) mass spectra were provided by the Engineering and Physical Science Research Council Mass Spectrometry Service in Swansea University.

### 3.2. 2′,3′-Di-O-benzyl-l-glycerol-(1′→1)-2-O-benzoyl-α-d-arabinofuranoside (**5**)

Tetrabutylammonium fluoride (17.8 mL, 0.0616 mol, 1.0 M in THF) was added dropwise with stirring to 2′,3′-di-*O*-benzyl-l-glycerol-(1′→1)-2-*O*-benzoyl-3,5-*O*-(tetraisopropylsiloxane-1,3-diyl)-α-d-arabinofuranoside (**7**) [20] (6.7 g, 0.0089 mol) in anhydrous THF (50 mL) at 0 °C under nitrogen. The mixture was allowed to reach room temperature (rt) and was stirred for 4 h, and it was then diluted with EtOAc (100 mL) and water (50 mL). The aqueous layer was re-extracted with EtOAc (3 × 50 mL). The combined organic layers were washed with sat. aq. NH_4_Cl (50 mL), brine (50 mL), dried and concentrated. Column chromatography eluting with petrol/EtOAc (1:1) gave compound (**5**) as a colourless thick oil (4.3 g, 95%) [MALDI: Found (M + Na)^+^: 531.2; C_29_H_32_NaO_8_ requires: 531.2], ${\left[\alpha \right]}_{D}^{23}$ +53 (*c* 4.0, CHCl_3_), which showed δ_H_ (400 MHz, CDCl_3_): 7.98–7.93 (2H, m), 7.53 (1H, t, *J* 7.4 Hz), 7.39 (2H, t, *J* 7.7 Hz), 7.33–7.17 (10H, m), 5.20 (1H, br. s), 5.04 (1H, br. s), 4.65 (1H, d, *J* 12.0 Hz), 4.61 (1H, d, *J* 12.0 Hz), 4.51 (1H, d, *J* 12.2 Hz), 4.48 (1H, d, *J* 12.2 Hz), 4.10 (2H, br. s), 3.85 (1H, dd, *J* 5.6, 10.3 Hz), 3.80 (1H, m), 3.76 (1H, br. p, *J* 5.2 Hz), 3.72–3.66 (1H, m), 3.63 (1H, dd, *J* 4.3, 10.3 Hz), 3.60–3.52 (2H, including br. d, *J* 4.8 Hz at 3.58), 2.59–2.31 (2H, incl. 2 × OH groups); δ_C_ (101 MHz, CDCl_3_): 166.6, 138.3, 138.0, 133.6, 129.8, 129.0, 128.5, 128.4, 128.3, 127.8, 127.6, 105.3, 85.9, 84.2, 76.6, 76.4, 73.4, 72.2, 69.7, 67.1, 61.9; ν_max_: 3405, 3065, 3031, 2945, 2868,1715, 1465, 1105, 884, 712 cm^−1^.

### 3.3. 2′,3′-Di-O-benzyl-l-glycerol-(1′→1)-2,3-di-O-benzoyl-5-O-tert-butyldiphenylsilyl-α-d-arabinofuranosyl-(1→3)-[2,3-di-O-benzoyl-5-O-tert-butyldiphenylsilyl-α-d-arabinofuranosyl-(1→5)]-2-O-benzoyl-α-d-arabinofuranoside (**6**)

Molecular sieves 4 Å (1.5 g) was added with stirring to furanoside (**4**) [18] (16.7 g, 28.9 mmol) and furanoside (**5**) (4.2 g, 8.2 mmol) in dry CH_2_Cl_2_ (50 mL) at rt under nitrogen. The mixture was stirred for 30 min., then cooled to −36 °C and *N*-iodosucinimide (9.1 g, 0.037 mol) was added followed by silver triflate (2.1 g, 8.2 mmol). The mixture was stirred until it turned dark brown, quenched by the addition of triethylamine (2 mL) until it turned yellow, then diluted with CH_2_Cl_2_ (100 mL) and filtered through celite. The filtrate was evaporated under reduced pressure; column chromatography eluting with hexane/EtOAc (5:2) gave compound (**6**) as a colourless thick oil (13.0 g, 91%) [MALDI: Found (M + NH_4_)^+^: 1682.6679; C_99_H_104_NO_20_Si_2_ requires: 1682.6685] (Figure S3), ${\left[\alpha \right]}_{D}^{17}$ −1.4 (*c* 2.8, CHCl_3_); δ_H_ (400 MHz, CDCl_3_): 8.01–7.92 (10H, m), 7.70–7.67 (4H, m), 7.65–7.61 (4H, m), 7.58–7.53 (2H, m), 7.50–7.44 (3H, m), 7.41–7.21 (32H, m), 5.66–5.62 (2H, incl. br. d, *J* 4.8 Hz at 5.63), 5.61 (1H, br.s), 5.55 (1H, br.d, *J* 1.3 Hz), 5.51 (1H, br.d, *J* 0.9 Hz), 5.43 (1H, br.d, *J* 0.9 Hz), 5.31 (1H, br.s), 5.22 (1H, br.s), 4.68 (2H, br.s), 4.53 (1H, d, *J* 12.0 Hz), 4.49 (1H, d, *J* 12.0 Hz), 4.47 (1H, br.s), 4.40 (1H, dd, *J* 5.2, 9.7 Hz), 4.38–4.32 (2H, incl. br. dd, *J* 5.8, 10.5 Hz at 4.36), 4.04 (1H, dd, *J* 4.9, 11.4 Hz), 3.95–3.89 (5H, m), 3.85 (1H, dd, *J* 2.2, 11.6 Hz), 3.81 (1H, dd, *J* 5.1, 10.1 Hz), 3.68 (1H, dd, *J* 4.7, 10.4 Hz), 3.65–3.59 (2H, incl. br. t, *J* 4.7 Hz at 3.63), 1.00 (9H, s), 0.96 (9H, s); δ_C_ (101 MHz, CDCl_3_): 165.5, 165.4, 165.2, 165.1, 138.6, 138.3, 135.7, 135.6, 135.5, 133.3, 133.2, 133.15, 133.1, 133.0, 130.0, 129.9, 129.8, 129.75, 129.7, 129.6, 129.35, 129.3, 129.25, 129.2, 128.4, 128.35, 128.3, 128.25, 128.2, 127.8, 127.7, 127.6, 127.55, 127.5, 127.4, 126.3, 106.1, 105.2, 83.7, 83.4, 82.3, 82.2, 82.1, 81.8, 80.5, 77.2, 76.6, 73.3, 72.2, 70.1, 67.2, 66.1, 63.3, 26.7, 26.6, 19.3, 19.2; ν_max_: 3069, 3010, 2932, 2857, 1723, 1602, 1452, 1072, 706 cm^−1^.

### 3.4. 2′,3′-Di-O-benzyl-l-glycerol-(1′→1)-2,3-di-O-benzyl-α-d-arabinofuranosyl-(1→3) [2,3-di-O-benzyl-α-d-arabinofuranosyl-(1→5)]-2-O-benzyl-α-d-arabinofuranoside (**9**)

(a) Sodium methoxide (25 mL, 1M, in methanol) was added to a stirred solution of furanoside (**6**) (9.1 g, 5.4 mmol) in dry CH_2_Cl_2_:MeOH (1:1, 50 mL) at rt until a pH of 11 was obtained. The mixture was stirred for 90 min, then neutralized by the addition of acetic acid. The solvent was evaporated under reduced pressure to give an oil; column chromatography eluting with chloroform/methanol (1:1) gave 2′,3′-di-*O*-benzyl-L-glycerol-(1′→1)-5-*O*-tert-butyldiphenyl-silyl-α-d-arabino-furanosyl-(1→3)-[5-*O*-tert-butyldiphenylsilyl-α-d-arabinofuranosyl-(1→5)]-α-d-arabino-furanoside (**8**) as a colourless thick oil (5.1 g, 83%) [MALDI: Found (M + Na)^+^: 1167.4913; C_64_H_80_NaO_15_Si_2_ requires: 1167.4928], ${\left[\alpha \right]}_{D}^{17}$ +35 (*c* 6.7, CHCl_3_) which showed δ_H_ (400 MHz, CDCl_3_): 7.69–7.62 (7H, m), 7.51–7.18 (23H, m), 5.18 (1H, br.s), 5.12 (1H, br.s), 4.93 (1H, br. s), 4.67 (1H, d, *J* 12.1 Hz), 4.63 (1H, d, *J* 12.1 Hz), 4.55 (1H, d, *J* 12.1 Hz), 4.51 (1H, d, *J* 12.1 Hz), 4.18 (1H, br. d, *J* 3.4 Hz), 4.16 (1H, br. dd, *J* 3.7, 6.1 Hz), 4.12 (1H, br. d, *J* 3.8 Hz), 4.07 (2H, br. s), 4.03–3.98 (2H, br. m), 3.98–3.93 (2H, incl. br. d, *J* 2.0 Hz at 3.96), 3.80 (1H, dd, *J* 3.3, 8.8 Hz), 3.78–3.68 (4H, m), 3.67–3.64 (1H, m), 3.63–3.59 (2H, incl. br. dd, *J* 3.0, 8.1 Hz at 3.60), 3.57 (1H, dd, *J* 5.2, 9.9 Hz), 3.02–2.55 (7H, br. m), 1.05 (9H, s), 1.03 (9H, s); δ_C_ (101 MHz, CDCl_3_): 138.4, 138.2, 135.6, 135.5, 131.9, 131.8, 131.7, 131.6, 130.2, 130.1, 130.0, 128.4, 128.35, 128.3, 128.0, 127.9, 127.8, 127.7, 127.6, 127.55, 127.5, 108.6, 108.4, 108.3, 87.8, 87.4, 83.7, 82.3, 79.5, 78.8, 78.5, 77.8, 77.7, 76.7, 76.6, 73.3, 71.9, 69.7, 67.0, 66.0, 64.0, 63.8, 26.7, 26.6, 19.0, 18.9; ν_max_: 3418, 3071, 2933, 2858, 1454, 1053, 822 cm^−1^.

(b) A solution of the above α-D-rabinofuranoside (**8**) (5.0 g, 4.0 mmol) in dry DMF was added dropwise to a stirred suspension of NaH (1.0 g, 43 mmol) (60% *w*/*w*, dispersion in mineral oil) at rt under nitrogen. The mixture was stirred for 10 min then benzyl bromide (5.2 g, 3.6 mL, 30 mmol) in dry DMF (2 mL) was added, stirred at rt for 6 h, then quenched by the slow addition of methanol (2 mL), and water (10 mL). The aqueous layer was re-extracted with EtOAc (2 × 50 mL). The combined organic layers were washed with water (25 mL) and brine (25 mL), dried and evaporated under reduced pressure. Column chromatography eluting with petrol/EtOAc (5:1) gave 2′,3′-di-*O*-benzyl-l-glycerol-(1′→1)-2,3-di-*O*-benzyl-5-*O*-tert-butyldiphenylsilyl-α-d-arabinofurano-syl-(1→3)-[2,3-di-*O*-benzyl-5-*O*-tert-butyldiphenylsilyl-α-d-arabinofuranosyl-(1→5)]-2-*O*-benzyl-α-d-arabinofuranoside as a colourless thick oil (4.5 g, 65%) [MALDI: Found (M+Na)^+^: 1617.7; C_99_H_110_NaO_15_Si_2_ requires: 1617.7], ${\left[\alpha \right]}_{D}^{17}$ +43 (*c* 1.9, CHCl_3_) which showed δ_H_ (400 MHz, CDCl_3_): 7.71–7.57 (8H, m), 7.48–7.13 (47H, m), 5.18 (2H, br. d, *J* 2.2 Hz), 5.07 (1H, br. s), 4.70 (1H, d, *J* 12.0 Hz), 4.66 (1H, d, *J* 12.0 Hz), 4.58–4.50 (7H, m), 4.47 (2H, d, *J* 11.9 Hz), 4.46 (1H, d, *J* 11.9 Hz), 4.39 (1H, d, *J* 11.9 Hz), 4.38 (1H, d, *J* 11.9 Hz), 4.30 (1H, br.dd, *J* 2.8, 6.8 Hz), 4.18 (2H, br. m), 4.13–4.07 (4H, m), 4.06–3.99 (2H, incl. br. dd, *J* 4.2, 10.6 Hz at 4.03), 3.95 (1H, dd, *J* 4.7, 11.9 Hz), 3.87 (1H, dd, *J* 5.0, 10.5 Hz), 3.84–3.80 (2H, incl. br. dd, *J* 4.0, 11.0 Hz at 3.82), 3.79–3.74 (4H, m), 3.68–3.58 (3H, m), 1.03 (18H, s); δ_C_ (101 MHz, CDCl_3_): 138.6, 138.3, 138.2, 138.0, 137.9, 137.6, 137.5, 135.75, 135.67, 135.65, 135.6, 133.55, 133.5, 133.4, 133.3, 129.6, 129.55, 129.5, 128.4, 128.35, 128.3, 128.25, 128.2, 127.9, 127.85, 127.8, 127.75, 127.7, 127.65, 127.6, 127.55, 127.5, 127.45, 127.4, 106.6, 106.4, 105.4, 88.6, 88.5, 88.0, 83.0, 82.8, 82.3, 81.8, 81.3, 80.3, 73.4, 72.2, 72.0, 71.9, 71.8, 71.7, 71.6, 70.4, 67.0, 66.0, 63.4, 63.2, 26.8, 26.7, 19.3, 19.2; ν_max_: 3067, 3031, 2929, 2857, 1495,1455, 1111, 698 cm^−1^.

(c) TBAF (14.3 mL, 0.0493 mol, in 1.0 M THF) was added dropwise to a stirred solution of the above furanoside (3.8 g, 2.3 mmol) in anhydrous THF (25 mL) at 0 °C under nitrogen. The mixture was allowed to reach rt and stirred for 8 h then diluted with EtOAc (100 mL) washed with sat. aq. NH_4_Cl (50 mL) and brine (50 mL). The organic layer was dried and concentrated; column chromatography eluting with hexane/EtOAc (1:1) gave the title compound (**9**) as a colourless thick oil (2.3 g, 87%) [MALDI–Found (M+Na)^+^: 1141.5, C_67_H_74_NaO_15_ requires: 1141.5], ${\left[\alpha \right]}_{D}^{17}$ +53 (*c* 4.7, CHCl_3_) which showed δ_H_ (400 MHz, CDCl_3_): 7.34–7.16 (35H, m), 5.10 (1H, br. s), 5.07 (1H, br. s), 5.04 (1H, br. d, *J* 0.7 Hz), 4.64 (2H, br. s), 4.55–4.39 (11H, m), 4.31 (1H, d, *J* 11.7 Hz), 4.27 (1H, dd, *J* 3.8, 7.4 Hz), 4.23–4.17 (1H, m), 4.07 (1H, br. d, *J* 2.2 Hz), 4.06 (1H, br. dd, *J* 4.0, 7.6 Hz), 4.02 (1H, br. dd, *J* 2.2, 5.9 Hz), 3.98 (1H, br. dd, *J* 1.3, 3.8 Hz), 3.96 (1H, br. dd, *J* 1.2, 3.6 Hz), 3.88 (1H, br. dd, *J* 4.1, 12.3 Hz), 3.85 (1H, br. dd, *J* 3.2, 6.5 Hz), 3.82 (1H, d, *J* 5.2 Hz), 3.78 (1H, dd, *J* 3.7, 7.4 Hz), 3.76–3.72 (2H, m), 3.71–3.64 (2H, incl. br. dd, *J* 12.3, 2.4 Hz at 3.68), 3.61 (1H, dd, *J* 4.8, 7.3 Hz), 3.59–3.55 (3H, incl. br. d, *J* 5.1 Hz at 3.58), 3.53 (1H, dd, *J* 5.9, 12.3 Hz), 1.5 (2H, br s); δ_C_ (101 MHz, CDCl_3_): 138.5, 138.2, 137.7, 137.6, 137.5, 137.4, 137.2, 128.5, 128.45, 128.4, 128.35, 128.3, 128.0, 127.95, 127.9, 127.85, 127.8, 127.75, 127.7, 127.6, 127.5, 106.1, 106.0, 105.9, 88.7, 88.3, 87.4, 83.0, 82.9, 82.4, 81.9, 80.8, 79.8, 73.4, 72.3, 72.25, 72.2, 72.0, 71.9, 71.8, 70.2, 67.2, 64.8, 62.7; ν_max_: 3459, 3064, 3030, 2921, 2860, 1605, 1496, 1115, 820 cm^−1^.

### 3.5. 2′,3′-Di-O-benzyl-l-glycerol-(1′→1)-2,3-di-O-benzyl-5-O-methanesulfonyl-α-d-arabinofuranosyl-(1→3)-[2,3-di-O-benzyl-5-O-methanesulfonyl-α-d-arabinofuranosyl-(1→5)]-2-O-benzyl-α-d-arabinofuranoside (**10**)

Methanesulfonyl chloride (0.57 g, 0.40 mL, 0.0050 mol) and DMAP (0.05g, 0.43 mmol) were added to a stirred solution of furanoside (**9**) (0.56 g, 0.50 mmol) in dry pyridine (5 mL) under nitrogen at rt. After 16 h it was quenched by the addition of water (1 mL); the organic layer was diluted with CH_2_Cl_2_ (10 mL) then washed with 1M aq. HCl (2 × 10 mL), sat. aq. NaHCO_3_ (2 × 10 mL), dried and evaporated to give a thick oil; column chromatography eluting with petrol/EtOAc (3:1) gave the title compound (**10**) as a colourless thick oil (0.55 g, 87%) [MALDI: Found (M+Na)^+^: 1297.4; C_69_H_78_NaO_19_S_2_ requires: 1297.4], ${\left[\alpha \right]}_{D}^{17}$ +59 (*c* 0.60, CHCl_3_), which showed δ_H_ (400 MHz, CDCl_3_): 7.40–7.15 (35H, m), 5.16 (1H, br. s), 5.13 (1H, br. s), 5.07 (1H, br. s), 4.68 (2H, br. s), 4.57–4.43 (11H, m), 4.40 (1H, d, *J* 11.3 Hz), 4.38–4.34 (1H, m), 4.33 (1H, br.dd, *J* 3.9, 7.3 Hz), 4.30–4.26 (2H, incl. br. dd, *J* 3.4, 7.3 Hz at 4.29), 4.24 (1H, d, *J* 5.4 Hz), 4.22–4.12 (2H, m), 4.11–4.07 (2H, m), 4.02–3.97 (2H, m), 3.91–3.84 (4H, m), 3.80 (1H, br. p, *J* 4.8 Hz), 3.73 (1H, br. dd, *J* 1.5, 11.5 Hz), 3.67–3.59 (3H, incl. br. dd, *J* 4.8, 8.7 Hz at 3.64), 2.94 (3H, s), 2.89 (3H, s); δ_C_ (101 MHz, CDCl_3_): 138.5, 138.2, 137.4, 137.3, 137.2, 137.0, 128.6, 128.55, 128.5, 128.45, 128.4, 128.3, 128.1, 128.05, 128.0, 127.95, 127.9, 127.7, 127.65, 127.6, 127.55, 106.5, 106.1, 105.8, 88.3, 87.7, 87.5, 82.9, 82.8, 80.3, 80.2, 79.3, 79.2, 77.2, 73.4, 72.35, 72.3, 72.2, 72.1, 72.0, 71.9, 70.2, 68.8, 68.5, 67.2, 65.2, 37.6, 37.5; ν_max_: 3088, 3065, 3031, 2933, 2871, 1586, 1454, 1177, 745 cm^−1^.

### 3.6. l-Glycerol-(1′→1)-5-O-behenate-α-d-arabinofuranosyl-(1→3)-5-O-behenate-α-d-arabinofuranosyl-(1→5)-α-d-arabinofuranoside (**11**, R = H)

(a) CsHCO_3_ (0.076 g, 0.39 mmol) was added to a stirred solution of dimesylate (**10**) (0.05 g, 0.03 mmol) and behenic acid (0.03 g, 0.09 mmol) in dry DMF:THF (1:5, 1 mL) at rt under nitrogen. The mixture was stirred at 70 °C for 3 days, then diluted with EtOAc (25 mL) and water (5 mL). The aqueous layer was re-extracted with EtOAc (2 × 10 mL). The combined organic layers were washed with water (5 mL) and brine (5 mL), dried and evaporated under reduced pressure to give a thick oil; column chromatography eluting with petrol/EtOAc (3:1) gave 2′,3′-di-*O*-benzyl-l-glycerol-(1′→1)-2,3-di-*O*-benzyl-5-*O*-behenate-α-d-arabinofuranosyl-(1→3)-[2,3-di-*O*-benzyl-5-*O*-behenate-α-d-arabinofuranosyl-(1→5)]-2-*O*-benzyl-α-d-arabinofuranoside (**11**, R = Bn) as a colourless thick oil (55 mg, 80%) [MALDI: Found (M + Na)^+^: 1786.1; C_111_H_158_NaO_17_ requires: 1786.1], ${\left[\alpha \right]}_{D}^{22}$ +36 (*c* 1.0, CHCl_3_) which showed δ_H_ (400 MHz, CDCl_3_): 7.31–7.11 (35H, m), 5.09 (1H, br. s), 5.06 (1H, br. s), 4.97 (1H, br. s), 4.59 (2H, br. s), 4.51–4.35 (10H, m), 4.33 (1H, d, *J* 11.8 Hz), 4.26 (1H, d, *J* 11.8 Hz), 4.20 (1H, br. dd, *J* 3.3, 7.3 Hz), 4.12 (6H, br. m), 4.04 (1H, br. dd, *J* 2.8, 6.9 Hz), 4.00 (1H, br. d, *J* 2.5 Hz), 3.95–3.89 (2H, m), 3.84 (1H, dd, *J* 4.3, 11.8 Hz), 3.81–3.76 (2H, incl. br. dd, *J* 4.1, 8.5 Hz at 3.78), 3.75 (1H, br. d, *J* 3.4 Hz), 3.74–3.68 (1H, p, *J* 5.0 Hz), 3.66 (1H, br. dd, *J* 2.1, 11.7 Hz), 3.58–3.49 (3H, incl. br. q, *J* 4.6 Hz at 3.53), 2.17 (4H, t, *J* 7.6 Hz), 1.56–1.00 (76H, m), 0.81 (6H, t, *J* 6.8 Hz); δ_C_ (101 MHz, CDCl_3_): 173.6, 173.5, 138.6, 138.3, 137.7, 137.6, 137.5, 137.4, 137.3, 128.5, 128.4, 128.35, 128.3, 128.0, 127.95, 127.9, 127.85, 127.8, 127.75, 127.7, 127.65, 127.6, 127.5, 106.5, 106.2, 105.5, 88.3, 88.2, 88.1, 83.4, 83.3, 80.8, 80.3, 79.2, 78.9, 76.9, 73.4, 72.3, 72.2, 72.1, 72.0, 71.9, 71.7, 70.3, 67.1, 65.6, 63.3, 63.2, 34.1, 34.0, 31.9, 29.7, 29.6, 29.5, 29.4, 29.35, 29.3, 29.2, 24.8, 22.7, 14.1; ν_max_: 3064, 3031, 2923, 2853, 1740, 1718, 1455, 1066, 698 cm^−1^.

(b) A general procedure for debenzylation was used throughout: Palladium hydroxide on activated charcoal (Pd(OH)_2_-C/20% (1.1 fold by weight) was added to a stirred solution of the benzyl protected compound (0.010 mmol) in CH_2_Cl_2_:MeOH:THF (1:1:1.5, 2 mL) at rt under hydrogen. The mixture was stirred for 36 h then filtered through celite and the solvent was evaporated under reduced pressure to give a residue; column chromatography eluting with chloroform/methanol (10:1) afforded the desired compound. In this way, compound (**11**, R = H) was obtained as a colourless thick oil (29 mg, 82%) [MALDI: Found (M + Na)^+^: 1155.8; C_62_H1_116_NaO_17_ requires: 1155.8], ${\left[\alpha \right]}_{D}^{18}$ −21 (*c* 1.1, CHCl_3_), which showed δ_H_ (400 MHz, CDCl_3_+few drops CD_3_OD): 5.01 (1H, br. s), 4.97 (1H, br. s), 4.90 (1H, br. s), 4.28–4.20 (2H, incl. br. dd *J* 3.2, 11.7 Hz at 4.24), 4.19–4.13 (3H, incl. br. dd *J* 5.0, 11.8 Hz at 4.17), 4.12 (1H, br. d, *J* 4.3 Hz), 4.11–4.08 (1H, m), 4.04 (1H, br. q, *J* 5.5 Hz), 4.01–3.95 (3H, br. m), 3.94 (1H, dd, *J* 3.6, 11.5 Hz), 3.83–3.76 (3H, m), 3.73 (1H, br. dd, *J* 4.8, 10.1 Hz), 3.67–3.62 (1H, m), 3.62–3.57 (2H, incl. br. d, *J* 3.1 Hz at 3.60), 3.56–3.51 (1H, m), 2.30 (4H, t, *J* 7.6 Hz), 1.64–1.02 (83H, m), 0.83 (6 H, t, *J* 6.5 Hz); δ_C_ (101 MHz, CDCl_3_): 174.05, 174.0, 108.0, 107.7, 83.3, 83.0, 82.4, 81.8, 81.3, 80.7, 79.0, 77.6, 75.8, 69.9, 69.1, 66.4, 63.9, 63.7, 63.5, 34.0, 33.95, 31.8, 29.6, 29.5, 29.45, 29.4, 29.3, 29.25, 29.2, 29.1, 29.0, 24.8, 24.7, 22.6, 13.9.; ν_max_: 3374, 2920, 2852, 1730, 1723, 1180, 757 cm^−1^.

### 3.7. 12l-Glycerol-(1′→1)-5-O-(2R)-2-(1-hydroxy-16-((1S,2R)-2-((S)-20-methyl-19-oxooctatria-contyl)cyclopropyl)hexadecyl)hexacosanoate-α-d-arabinofuranosyl-(1→3)-[5-O-((2R)-2-(1-hydroxy-16-((1S,2R)-2-((S)-20-methyl-19-oxooctatriacontyl)cyclopropyl)hexadecyl)hexacosan-oate-α-d-arabinofuranosyl-(1→5)]-α-d-arabinofuranoside (**15a**)

(a) 1-Ethyl-3-(3-dimethylaminopropyl)carbodiimide hydrochloride (EDCI) (60 mg, 0.31 mmol), in dry CH_2_Cl_2_ (1 mL) was added to a stirred solution of α-D-arabinofuranoside (**9**) (35 mg, 0.031 mmol), molecular sieves 4 Å (50 mg), DMAP (38 mg, 0.31) (2*R*)-2-(1-((*tert*-butyldimethyl-silyl)oxy)-16-((1*S*,2*R*)-2-((*S*)-20-methyl-19-oxooctatriacontyl)cyclopropyl)hexadecyl)hexacosanoic acid (**12a**) (85 mg, 0.062 mmol) [30] in dry CH_2_Cl_2_ (1 mL) at rt under nitrogen. After 4 days, the precipitate was filtered off and washed with CH_2_Cl_2_ (10 mL), and the solvent was evaporated; column chromatography eluting with hexane/EtOAc (10:1) gave compound (**13a**) as a colourless thick oil (60 mg, 51%) [MALDI: Found (M + Na)^+^: 3808.2; C_247_H_426_NaO_21_Si_2_ requires: 3808.2], ${\left[\alpha \right]}_{D}^{20}$ +18 (*c* 5.0, CHCl_3_), which showed δ_H_ (400 MHz, CDCl_3_): 7.69–6.95 (35H, m), 5.16 (1H, br. s), 5.13 (1H, br. s), 5.04 (1H, br. s), 4.68 (1H, d, *J* 12.1 Hz), 4.65 (1H, d, *J* 12.1 Hz), 4.58–4.45 (9H, m), 4.45 (1H, d, *J* 11.9 Hz), 4.38 (1H, d, *J* 11.9 Hz), 4.33 (1H, d, *J* 11.8 Hz), 4.29–4.22 (5H, incl. br. dd *J* 4.0, 9.1 Hz at 4.25), 4.21–4.16 (2H, m), 4.13 (1H, br. dd, *J* 5.7, 8.2 Hz), 4.06 (1H, br. d, *J* 2.7 Hz), 4.01–3.96 (2H, m), 3.94–3.87 (5H, m), 3.85 (1H, br. dd, *J* 4.8, 10.7 Hz), 3.78 (1H, br. p, *J* 5.1 Hz), 3.76–3.71 (1H, m), 3.65–3.56 (3H, incl. br. dd, *J* 4.8, 8.2 Hz at 3.60), 2.53 (4H, incl. sextet, *J* 5.4 Hz), 2.42 (4H, t, *J* 7.6 Hz), 1.68–1.11 (288H, m), 1.06 (6H, d, *J* 6.9 Hz), 0.89 (12H, t, *J* 6.8 Hz), 0.85 (9H, s), 0.84 (9H, s), 0.71–0.61 (4H, m), 0.57 (2H, dt, *J* 4.0, 8.5 Hz), 0.03 (6H, s), 0.01 (6H, s), −0.32 (2H, br.q, *J* 5.1 Hz); δ_C_ (101 MHz, CDCl_3_): 215.2, 174.3, 174.2, 138.5, 138.2, 137.8, 137.7, 137.6, 137.4, 137.3, 128.5, 128.45, 128.4, 128.35, 128.3, 128.2, 127.9, 127.85, 127.8, 127.75, 127.7, 127.65, 127.6, 127.55, 127.5, 106.6, 106.3, 105.2, 88.3, 88.2, 87.8, 83.7, 83.6, 81.2, 80.2, 79.4, 78.9, 77.2, 73.4, 73.1, 72.3, 72.2, 72.0, 71.9, 71.6, 70.3, 67.0, 65.9, 63.0, 62.7, 51.6, 46.3, 41.1, 33.6, 33.5, 33.0, 31.9, 30.3, 30.2, 29.9, 29.8, 29.75, 29.7, 29.65, 29.6, 29.55, 29.5, 29.45, 29.4, 29.3, 28.7, 27.9, 27.8, 27.3, 27.2, 27.1, 25.8, 24.0, 23.7, 22.7, 22.6, 18.0, 16.4, 15.8, 14.1, 10.9, −4.4, −4.5, −4.7, −4.8; ν_max_: 3063,3031, 2920, 2851, 1741, 1714, 1467, 1106, 836, 699 cm^−1^.

(b) The protected furanoside (**13a**) (53 mg, 0.014 mmol) was dissolved in dry THF (10 mL) in a dry polyethylene vial equipped with an acid-proof rubber septum, followed by the addition of pyridine (0.1 mL) at rt under nitrogen. The mixture was cooled to 0 °C, and then HF–pyridine complex (70% w, 1.5 mL) was added dropwise. The mixture was stirred at 43 °C for 24 h, then poured slowly into sat. aq. NaHCO_3_ and stirred until no more CO_2_ was liberated. The aqueous layer was re-extracted with chloroform (3 × 10 mL). The combined organic layers were dried and evaporated. Column chromatography eluting with hexane/EtOAc (10:1) afforded compound (**14a**) as a colourless thick oil (38 mg, 76%) [MALDI: Found (M+Na)^+^: 3579.9; C_235_H_398_NaO_21_ requires: 3579.9], ${\left[\alpha \right]}_{D}^{21}$ +19 (*c* 1.2, CHCl_3_); δ_H_ (400 MHz, CDCl_3_): 7.36–7.15 (35H, m), 5.16 (1H, br. s), 5.13 (1H, br. s), 5.05 (1H, br. s), 4.67 (2H, br. s), 4.55 (1H, d, *J* 11.8 Hz), 4.53–4.47 (7H, m), 4.45 (3H, d, *J* 11.8 Hz), 4.40 (1H, d, *J* 11.8 Hz), 4.36–4.22 (7H, m), 4.17 (1H, br. dd, *J* 3.7, 6.9 Hz), 4.11 (1H, br. dd, *J* 3.3, 6.3 Hz), 4.07 (1H, br. d, *J* 2.1 Hz), 4.00–3.96 (2H, m), 3.94–3.88 (2H, m), 3.88–3.82 (2H, incl. br. dd *J* 4.5, 9.8Hz at 3.86), 3.81–3.75 (2H, m), 3.73 (1H, br. d, *J* 11.7 Hz), 3.67–3.51 (5H, incl. br. d *J* 5.3 Hz at 3.61), 2.51 (2H, sextet, *J* 6.7 Hz), 2.42 (6H, incl. t *J* 7.2 Hz), 2.05–1.10 (288H, m), 1.06 (6H, d, *J* 6.9 Hz), 0.89 (12H, t, *J* 6.7 Hz), 0.69–0.61 (4H, m), 0.57 (2H, dt, *J* 4.1, 8.5 Hz), -0.33 (2H, br. q, *J* 5.0 Hz); δ_C_ (101 MHz, CDCl_3_): 215.2, 175.1, 175.0, 138.6, 138.2, 137.6, 137.5, 137.45, 137.4, 137.2, 128.5, 128.4, 128.35, 128.3, 128.2, 128.1, 128.0, 127.95, 127.9, 127.85, 127.8, 127.75, 127.7, 127.6, 127.5, 106.3, 106.2, 105.5, 88.2, 88.0, 87.9, 83.6, 80.7, 80.3, 79.3, 79.2, 77.2, 73.4, 72.4, 72.2, 72.1, 72.0, 71.7, 70.2, 68.0, 67.1, 65.4, 63.0, 62.9, 51.9, 51.7, 46.3, 41.1, 35.3, 35.2, 33.0, 31.9, 30.3, 30.2, 29.8, 29.7, 29.65, 29.6, 29.55, 29.5, 29.45, 29.4, 29.3, 29.2, 28.7, 27.5, 27.4, 27.3, 25.7, 25.6, 23.7, 22.7, 16.4, 15.8, 14.1, 10.9; ν_max_: 3511, 3063,3030, 2918, 2851, 1737, 1714, 1467, 1105, 754, 698 cm^−1^.

(c) Using the generalised procedure for debenzylation, compound (**15a**) was obtained as a colourless thick oil (25 mg, 86%) [MALDI: Found (M + Na)^+^: 2949.7; C_186_H_356_NaO_21_ requires: 2949.7], ${\left[\alpha \right]}_{D}^{18}$ +19 (*c* 2.2, CHCl_3_); δ_H_ (400 MHz, CDCl_3_ + few drops CD_3_OD): 5.00 (1H, br. s), 4.96 (1H, br. s), 4.90 (1H, br. s), 4.41 (1H, dd, *J* 4.2, 11.6 Hz), 4.36 (1H, dd, *J* 5.1, 11.9 Hz), 4.26 (1H, dd, *J* 11.9, 5.4 Hz), 4.21 (1H, d, *J* 4.0 Hz), 4.17 (1H, dd, *J* 3.8, 10.3 Hz), 4.12 (1H, br. d, *J* 4.8 Hz), 4.08 (1H, br. q, *J* 6.6 Hz), 4.04 (1H, d, *J* 5.1 Hz), 4.01–3.96 (3H, m), 3.92 (1H, dd, *J* 3.8, 8.0 Hz), 3.90–3.86 (2H, m), 3.77 (1H, dd, *J* 2.7, 8.4 Hz), 3.72 (1H, d, *J* 5.1 Hz), 3.62 (5H, br. m), 3.54 (1H, dd, *J* 2.6, 10.5 Hz), 2.52–2.44 (2H, m), 2.38 (6H, incl. t, *J* 7.5 Hz), 1.65–1.05 (297 H, m), 1.01 (6H, d, *J* 6.9 Hz), 0.84 (12H, t, *J* 6.8 Hz), 0.66–0.57 (4H, m), 0.52 (2H, dt, *J* 4.2, 8.2 Hz), -0.37 (2H, br. q, *J* 4.7 Hz); δ_C_ (101 MHz, CDCl_3_): 216.2, 175.05, 174.9, 109.2, 108.7, 81.95, 81.2, 79.4, 78.0, 77.6, 77.2, 76.5, 72.5, 71.9, 65.0, 63.5, 63.4, 61.5, 52.6, 35.0, 32.8, 31.8, 30.1, 29.6, 29.55, 29.5, 29.4, 29.3, 29.25, 29.2, 28.9, 28.6, 27.3, 27.2, 26.1, 25.3, 23.5, 22.6, 16.1, 15.7, 14.0, 10.8.; ν_max_: 3511, 3063,3030, 2918, 2851, 1737, 1714, 1467, 1105, 754, 698 cm^−1^.

### 3.8. l-Glycerol-(1′→1)-5-O-(2R)-2-((1R)-1-hydroxy-12-((2R)-2-(14-((2R)-2-eicosylcyclo-propyl)tetradecyl)cyclopropyl)dodecyl)hexacosanoate-α-d-arabinofuranosyl-(1→3)-[5-O-(2R)-2-((1R)-1-hydroxy-12-((2R)-2-(14-((2R)-2-icosylcyclopropyl)tetradecyl)cyclopropyl)-dodecyl)hexacosanoate-α-d-arabinofuranosyl-(1→5)]-α-d-arabinofuranoside (**15b**)

(a) A solution of EDCI (48 mg, 0.25 mmol) in dry CH_2_Cl_2_ (1 mL) was added with stirring to furanoside (**9**) (28 mg, 0.025 mmol), molecular sieves 4 Å (50 mg), DMAP (30 mg, 0.24 mmol) and (2*R*)-2-((1*R*)-1-((*tert*-butyldimethylsilyl)oxy)-12-((2*R*)-2-(14-((2*R*)-2-eicosylcyclopropyl)tetradecyl)cyclopropyl)-dodecyl)hexacosanoic acid (**12b**) (62 mg, 0.049 mmol) [31] in dry CH_2_Cl_2_ (1 mL) at rt under nitrogen. After 4 days, the precipitate was filtered and washed with CH_2_Cl_2_ (10 mL), the solvent was evaporated and the residue was purified by column chromatography eluting with hexane/EtOAc (10:1) to afford compound (**13b**) as a colourless thick oil (75 mg, 84%) [MALDI: Found (M+Na)^+^: 3607.9; C_235_H_402_NaO_19_Si_2_ requires: 3607.9], ${\left[\alpha \right]}_{D}^{22}$ +20 (*c* 0.90, CHCl_3_), which showed δ_H_ (400 MHz, CDCl_3_): 7.94–6.85 (35H, m), 5.16 (1H, br. s), 5.13 (1H, br. s), 5.04 (1H, br. s), 4.68 (1H, d, *J* 12.1 Hz), 4.65 (1H, d, *J* 12.1 Hz), 4.57–4.45 (9H, m), 4.45 (1H, d, *J* 11.8 Hz), 4.38 (1H, d, *J* 11.8 Hz), 4.32 (1H, d, *J* 11.8 Hz), 4.28–4.20 (5H, m), 4.18 (1H, br. dd, *J* 3.6, 7.4 Hz), 4.15–4.09 (1H, m), 4.06 (1H, br. d, *J* 2.9 Hz), 4.01–3.96 (2H, m), 3.90 (3H, br. m), 3.87–3.81 (2H, incl. br. dd, *J* 4.8, 10.7 Hz at 3.85), 3.78 (1H, br. p, *J* 5.1 Hz), 3.76–3.71 (1H, m), 3.64–3.49 (3H, incl. br. dd, *J* 4.8, 8.3 Hz at 3.60), 2.60–2.47 (2H, m), 1.67–1.06 (270H, m), 0.89 (12H, t, *J* 6.8 Hz), 0.85 (9H, s), 0.84 (9H, s), 0.74–0.62 (8H, m), 0.57 (4H, td, *J* 4.1 Hz), 0.02 (6H, s), 0.00 (6H, s), −0.33 (4H, br. q, *J* 4.9 Hz); δ_C_ (101 MHz, CDCl_3_): 174.3, 174.2, 138.5, 138.3, 137.8, 137.7, 137.6, 137.4, 137.3, 128.5, 128.45, 128.4, 128.35, 128.3, 127.9, 127.85, 127.8, 127.75, 127.7, 127.65, 127.6, 127.55, 127.5, 106.6, 106.3, 105.2, 88.3, 88.2, 87.8, 83.7, 83.6, 81.2, 80.2, 79.4, 78.9, 77.2, 73.4, 73.1, 72.3, 72.2, 71.9, 71.6, 70.3, 67.0, 65.9, 63.0, 62.7, 51.6, 33.6, 33.5, 31.9, 30.3, 30.2, 29.9, 29.8, 29.7, 29.6, 29.5, 29.4, 28.7, 27.9, 27.8, 27.2, 27.1, 25.9, 25.8, 24.0, 22.7, 18.0, 15.8, 14.1, 11.0, 10.9, −4.4, −4.5, −4.7, −4.8; ν_max_: 3063, 2925, 2854, 1737, 1456, 1101, 770 cm^−1^.

(b) The protected glycolipid α-d-arabinofuranoside (**13b**) (70 mg, 0.019 mmol) was dissolved in dry THF (10 mL) in a dry polyethylene vial equipped with an acid-proof rubber septum, followed by the addition of pyridine (0.1 mL) at rt under nitrogen. The mixture was cooled to 0 °C, and then HF–pyridine complex (70% w, 1.5 mL) was added dropwise. The mixture was stirred at 43 °C for 24 h, then poured slowly into sat. aq. NaHCO_3_ and stirred until no more CO_2_ was liberated. The aqueous layer was re-extracted with chloroform (3 × 10 mL). The combined organic layers were dried and evaporated; column chromatography eluting with hexane/EtOAc (10:1) afforded compound (**14b**) as a colourless thick oil (33 mg, 51%) [MALDI: Found (M+Na)^+^: 3379.8; C_223_H_374_NaO_19_ requires: 3379.8], ${\left[\alpha \right]}_{D}^{21}$ +22 (*c* 0.90, CHCl_3_), which showed δ_H_ (400 MHz, CDCl_3_): 7.37–7.17 (35H, m), 5.16 (1H, br. s), 5.13 (1H, br. s), 5.05 (1H, br. s), 4.67 (2H, br. s), 4.55 (1H, d, *J* 11.9 Hz), 4.53–4.47 (8H, m), 4.46 (2H, d, *J* 11.8 Hz), 4.41 (1H, d, *J* 11.9 Hz), 4.35–4.23 (6H, m), 4.17 (1H, br. dd, *J* 3.7, 6.9 Hz), 4.15–4.08 (3H, incl. br. t *J* 7.0 Hz at 4.12), 4.07 (1H, br. d, *J* 2.1 Hz), 4.00–3.95 (2H, m), 3.94–3.87 (2H, m), 3.95–3.87 (2H, incl. br. dd, *J* 4.6, 10.3 Hz at 3.86), 3.79 (1H, p, *J* 4.8 Hz), 3.75–3.69 (1H, m), 3.67–3.55 (3H, incl. br. d, *J* 5.3 Hz at 3.61), 2.44–2.37 (2H, m), 1.67–1.03 (270H, m), 0.89 (12H, t, *J* 7.1 Hz), 0.69–0.61 (8H, m), 0.57 (4H, dt, *J* 4.2, 8.5 Hz), −0.32 (4H, br. q, *J* 4.9 Hz); δ_C_ (101 MHz, CDCl_3_): 175.1, 175.0, 138.5, 138.2, 137.6, 137.5, 137.4, 137.4, 137.2, 128.5, 128.4, 128.35, 128.3, 128.0, 127.95, 127.9, 127.85, 127.8, 127.75, 127.7, 127.6, 127.5, 106.3, 106.2, 105.5, 88.2, 88.0, 87.9, 83.6, 80.7, 80.3, 79.3, 79.2, 77.2, 73.4, 72.4, 72.2, 72.0, 71.9, 71.7, 70.3, 67.1, 65.4, 63.1, 63.0, 62.0, 51.9, 51.8, 35.3, 35.2, 34.2, 31.9, 30.3, 30.2, 29.8, 29.75, 29.7, 29.65, 29.6, 29.5, 29.4, 28.7, 27.5, 27.4, 25.8, 25.7, 24.9, 22.7, 22.6, 20.4, 15.8, 14.1, 10.9; ν_max_: 3511, 3062, 2921, 2854, 1733,1725, 1456, 1115, 754 cm^−1^.

(c) Using the generalised debenzylation procedure, compound (**15b**) was obtained as a colourless thick oil (22 mg, 88%) [MALDI: Found (M+Na)^+^: 2749.5; C_174_H_332_NaO_19_ requires: 2749.5], ${\left[\alpha \right]}_{D}^{18}$ +18 (*c* 2.2, CHCl_3_), which showed δ_H_ (400 MHz, CDCl_3_+few drops CD_3_OD): 5.00 (1H, br. s), 4.96 (1H, br. s), 4.90 (1H, br. s), 4.41 (1H, dd, *J* 4.6, 11.7 Hz), 4.37 (1H, br. dd, *J* 5.1, 11.9 Hz), 4.26 (1H, dd, *J* 5.0, 11.7 Hz), 4.20 (1H, dd, *J* 4.7, 11.8 Hz), 4.16 (1H, br. q, *J* 4.8 Hz), 4.12 (1H, br. d, *J* 6.2 Hz), 4.10–4.06 (2H, incl. br. t, *J* 7.4 Hz at 4.08), 4.05 (1H, br. d, *J* 5.6 Hz), 4.02–3.96 (3H, incl. br. d, *J* 8.5 Hz at 3.99), 3.95–3.86 (3H, m), 3.77 (1H, dd, *J* 4.2, 7.4 Hz), 3.72 (1H, d, *J* 5.4 Hz), 3.67–3.57 (4H, incl. br. d, *J* 10.5 Hz at 3.63), 3.54 (1H, dd, *J* 2.6, 9.7 Hz), 2.42–2.35 (2H, m), 1.64–1.04 (277H, m), 0.84 (12H, t, *J* 6.7 Hz), 0.64–0.56 (8H, m), 0.52 (4H, dt, *J* 4.1, 8.4 Hz), −0.37 (4H, br. q, *J* 4.9 Hz); δ_C_ (101 MHz, CDCl_3_+ few drops CD_3_OD): 175.0, 174.9, 108.7, 108.0, 81.7, 81.3, 79.7, 79.4, 77.9, 77.5, 77.2, 76.3, 72.4, 71.9, 69.7, 69.15, 68.9, 67.6, 65.1, 63.4, 62.2, 61.4, 57.8, 52.6, 34.9, 31.8, 30.1, 29.55, 29.5, 29.45, 29.4, 29.3, 29.2, 29.15, 29.1, 28.6, 27.3, 25.3, 22.5, 20.5, 15.6, 13.9, 10.7.; ν_max_: 3399, 2920, 2851, 1734, 1467, 1043 cm^−1^. 

### 3.9. l-Glycerol-(1′→1)-5-O-(2R)-2-((1R)-1-hydroxy-17-((1S,2R)-2-((22S)-22-methyl-21-oxotetracontan-2-yl)cyclopropyl)heptadecyl)hexacosanoate-α-d-arabinofuranosyl-(1→3)-[-5-O-(2R)-2-((1R)-1-hydroxy-17-((1S,2R)-2-((22S)-22-methyl-21-oxotetracontan-2-yl)cyclo-propyl)heptadecyl)hexacosanoate-α-d-arabinofuranosyl-(1→5)]-α-d-arabinofuranoside (**15c**)

(a) A solution of EDCI (35 mg, 0.18 mmol) in dry CH_2_Cl_2_ (1 mL) was added to a stirred solution of α-D-arabinofuranoside (**9**) (22 mg, 0.019 mmol), molecular sieves 4 Å (25 mg), DMAP (23 mg, 0.18 mmol) and (2*R*)-2-((1*R*)-1-((*tert*-butyldimethylsilyl)oxy)-17-((1*S*,2*R*)-2-((22*S*)-22-methyl-21-oxotetra-contan-2yl)cyclopropyl)-heptadecyl)hexacosanoic acid (**12c**) (50 mg, 0.035 mmol) [32] in dry CH_2_Cl_2_ (1 mL) at rt under nitrogen. The mixture was stirred for 4 days, then the precipitate was filtered off and washed with CH_2_Cl_2_ (10 mL), the solvent was evaporated and the residue was purified by column chromatography eluting with hexane/EtOAc (10:1) to afford the title compound (**13c**) as a colourless thick oil (35 mg, 46%) [MALDI: Found (M + Na)^+^: 3892.2; C_253_H_438_NaO_21_Si_2_ requires: 3892.2], ${\left[\alpha \right]}_{D}^{20}$ +14 (*c* 3.0, CHCl_3_), which showed δ_H_ (400 MHz, CDCl_3_): 7.54–7.00 (35H, m), 5.16 (1H, br. s), 5.13 (1H, br. s), 5.04 (1H, br. s), 4.68 (1H, d, *J* 12.1 Hz), 4.65 (1H, d, *J* 12.1 Hz), 4.58–4.47 (9H, m), 4.46 (1H, d, *J* 11.9 Hz), 4.39 (1H, d, *J* 11.8 Hz), 4.33 (1H, d, *J* 11.9 Hz), 4.30–4.22 (5H, incl. br. dd, *J* 3.9, 10.4 Hz at 4.26), 4.22–4.16 (2H, m), 4.13 (1H, br. dd, *J* 5.6, 8.4 Hz), 4.06 (1H, br. d, *J* 2.9 Hz), 4.01–3.97 (2H, m), 3.95–3.87 (5H, m), 3.85 (1H, br. dd, *J* 3.8, 9.4 Hz), 3.78 (1H, br. p, *J* 4.9 Hz), 3.76–3.72 (1H, m), 3.65–3.55 (3H, incl. br. dd *J* 4.8, 8.3 Hz at 3.60), 2.53 (4H, incl. sextet, *J* 5.6 Hz), 2.42 (4H, t, *J* 7.5 Hz), 1.62–1.14 (292 H, m), 1.06 (6H, d, *J* 6.9 Hz), 0.89 (12H, t, *J* 6.8 Hz), 0.88 (6H, d, *J* 6.8 Hz), 0.85 (9H, s), 0.84 (9H, s), 0.75–0.62 (2H, m), 0.51–0.40 (2H, m), 0.24–0.08 (6H, m), 0.03 (6H, s), 0.01 (6H, s); δ_C_ (101 MHz, CDCl_3_): 215.1, 174.2, 174.1, 138.6, 138.3, 137.8, 137.7, 137.6, 137.55, 137.5, 128.45, 128.4, 128.35, 128.3, 128.25, 128.2, 127.9, 127.85, 127.8, 127.75, 127.7, 127.65, 127.6, 127.55, 127.5, 106.6, 106.3, 105.2, 88.3, 88.2, 87.8, 83.8, 83.7, 81.2, 80.2, 79.5, 78.9, 77.2, 73.4, 73.2, 72.3, 72.2, 72.0, 71.9, 71.6, 70.3, 67.0, 65.9, 63.0, 62.7, 51.6, 46.3, 41.1, 38.1, 37.4, 34.5, 33.7, 33.6, 33.0, 31.9, 30.1, 29.9, 29.8, 29.75, 29.7, 29.65, 29.6, 29.55, 29.5, 29.45, 29.4, 29.35, 29.3, 27.8, 27.4, 27.3, 27.2, 27.1, 26.1, 25.9, 25.8, 24.1, 23.7, 22.7, 22.6, 19.7, 18.6, 18.0, 16.4, 14.1, 10.5, −4.4, −4.5, −4.7, −4.8; ν_max_: 3063,3032, 2919, 2851, 1739, 1714, 1467, 1105, 836, 698 cm^−1^.

(b) The protected glycolipid furanoside (**13c**) (31 mg, 0.0080 mmol) was dissolved in dry THF (10 mL) in a dry polyethylene vial equipped with an acid-proof rubber septum, followed by the addition of pyridine (0.1 mL) at rt under nitrogen. The mixture was cooled to 0 °C, and then HF–pyridine complex as (70% w, 1.5 mL) was added dropwise. The mixture was stirred at 43 °C for 24 h, then poured slowly into sat. aq. NaHCO_3_ and stirred until no more CO_2_ was liberated. The aqueous layer was re-extracted with chloroform (3 × 10 mL). The combined organic layers were dried and evaporated; column chromatography eluting with hexane/EtOAc (10:1) afforded compound (**14c**) as a colourless thick oil (19 mg, 66%) [MALDI: Found (M + Na)^+^: 3664.1; C_241_H_410_NaO_21_ requires: 3664.1], ${\left[\alpha \right]}_{D}^{18}$ +25 (*c* 1.1, CHCl_3_), which showed δ_H_ (400 MHz, CDCl_3_): 7.39–7.18 (35H, m), 5.16 (1H, br. s), 5.13 (1H, br. s), 5.04 (1H, br. s), 4.67 (2H, br. s), 4.55 (1H, d, *J* 11.9 Hz), 4.53–4.47 (8H, m), 4.45 (2H, d, *J* 11.8 Hz), 4.40 (1H, d, *J* 11.8 Hz), 4.29 (6H, br. m), 4.17 (1H, br. dd, *J* 3.8, 6.9 Hz), 4.11 (1H, br. dd, *J* 3.4, 6.2 Hz), 4.07 (1H, br. d, *J* 2.5 Hz), 3.99–3.95 (2H, m), 3.94–3.87 (2H, m), 3.87–3.82 (2H, incl. br. dd, *J* 4.6, 10.3 Hz at 3.85), 3.79 (1H, br. p, *J* 4.9 Hz), 3.72 (1H, br.dd, *J* 1.7, 11.6 Hz), 3.64–3.57 (5H, m), 2.51 (2H, sextet, *J* 6.8 Hz), 2.42 (6H, incl. t, *J* 7.5 Hz), 1.87–1.08 (300H, m), 1.05 (6H, d, *J* 6.9 Hz), 0.89 (12H, t, *J* 7.3 Hz), 0.72–0.62 (2H, m), 0.50–0.38 (2H, m), 0.25–0.05 (6H, m); δ_C_ (101 MHz, CDCl_3_): 215.2, 175.1, 175.0, 138.6, 138.3, 137.7, 137.5, 137.45, 137.4, 137.2, 128.5, 128.4, 128.35, 128.3, 128.2, 128.0, 127.95, 127.9, 127.85, 127.8, 127.75, 127.7, 127.6, 106.3, 106.2, 105.4, 88.2, 88.0, 87.9, 83.6, 80.7, 80.3, 79.3, 79.2, 77.2, 73.4, 72.4, 72.2, 72.1, 72.0, 71.9, 71.7, 70.3, 67.1, 65.4, 63.0, 62.9, 51.9, 51.7, 46.3, 41.1, 38.1, 37.4, 35.3, 35.2, 34.5, 33.0, 31.9, 30.1, 29.75, 29.7, 29.65, 29.6, 29.55, 29.5, 29.45, 29.4, 29.3, 27.5, 27.4, 27.35, 27.3, 26.1, 25.7, 23.7, 22.7, 19.7, 18.6, 16.4, 14.1, 10.5; ν_max_: 3457, 3063,3031, 2919, 2852, 1737, 1715, 1464, 1104,734, 698 cm^−1^.

(c) Using the generalised debenzylation procedure, compound (**15c**) was obtained as a colourless oil (9.0 mg, 82%) [MALDI: Found (M+Na)^+^: 3033.8; C_192_H_368_NaO_21_ requires: 3033.8], ${\left[\alpha \right]}_{D}^{18}$ +26 (*c* 0.90, CHCl_3_); δ_H_ (400 MHz, CDCl_3_+ few drops CD_3_OD): 5.01 (1H, br. s), 4.96 (1H, br. s), 4.91 (1H, br. s), 4.42 (1H, dd, *J* 4.9, 12.1 Hz), 4.37 (1H, dd, *J* 4.6, 11.8 Hz), 4.27 (1H, dd, *J* 4.7, 11.1Hz), 4.22 (1H, br. d, *J* 4.7 Hz), 4.18 (1H, dd, *J* 4.2, 9.0 Hz), 4.13 (1H, br. q, *J* 5.4 Hz), 4.09 (1H, t, *J* 7.0 Hz), 4.06 (1H, d, *J* 5.1 Hz), 4.02–3.97 (3H, br. m), 3.93 (1H, br. dd, *J* 3.3, 7.3 Hz), 3.89 (2H, br. m), 3.80–3.72 (2H, m), 3.67–3.58 (5H, m), 3.55 (1H, dd, *J* 2.4, 9.6 Hz), 2.53–2.44 (2H, sextet, *J* 6.6 Hz), 2.39 (6H, incl. t, *J* 7.4 Hz), 1.57–1.06 (210 H, m), 1.01 (6H, d, *J* 6.9 Hz), 0.85 (12H, t, *J* 7.5 Hz), 0.67–0.57 (2H, m), 0.47–0.34 (2H, m), 0.21–0.02 (6H, m); δ_C_ (101 MHz, CDCl_3_): 215.8, 175.0, 174.75, 109.3, 108.7, 87.1, 82.95, 82.9, 80.9, 79.4, 78.8, 78.2, 77.9, 77.2, 72.6, 71.7, 68.8, 65.0, 64.0, 63.3, 61.8, 52.5, 46.3, 41.1, 38.1, 37.4, 35.1, 34.4, 33.0, 32.9, 31.9, 30.0, 29.7, 29.6, 29.55, 29.5, 29.45, 29.4, 29.35, 29.3, 27.4, 27.35, 27.3, 27.25, 27.2, 26.1, 25.45, 25.4, 23.6, 23.55, 22.6, 19.6, 18.6, 16.3, 16.2, 14.1, 10.4.; ν_max_: 3434, 2919, 2852, 1736, 1717, 1467, 1104,735, 699 cm^−1^.

## Supplementary Materials

Supplementary data related to this article, including the Figures S1–S7 and Table S1 referred to in the text, can be found online at https://www.mdpi.com/1420-3049/24/19/3596/s1.
