# Supplementary material for: Synthesis of a Di-Mycoloyl Tri-Arabinofuranosyl Glycerol Fragment of the Mycobacterial Cell Wall, Based on Synthetic Mycolic Acids
Source: Molecules. 2019 Oct 6;24(19):3596. doi: 10.3390/molecules24193596 (PMC6804083; doi:10.3390/molecules24193596)
Supplement: Supplementary file 1 [file molecules-24-03596-s001.pdf]

Supporting Information

Synthesis of di-mycoloyl tri-arabinofuranosyl glycerol fragment of mycobacterial cell wall based on synthetic mycolic acids

Omar T. Ali, Mohsin O. Mohammed, Juma'a R. Al Dulayymi, Mark S. Baird

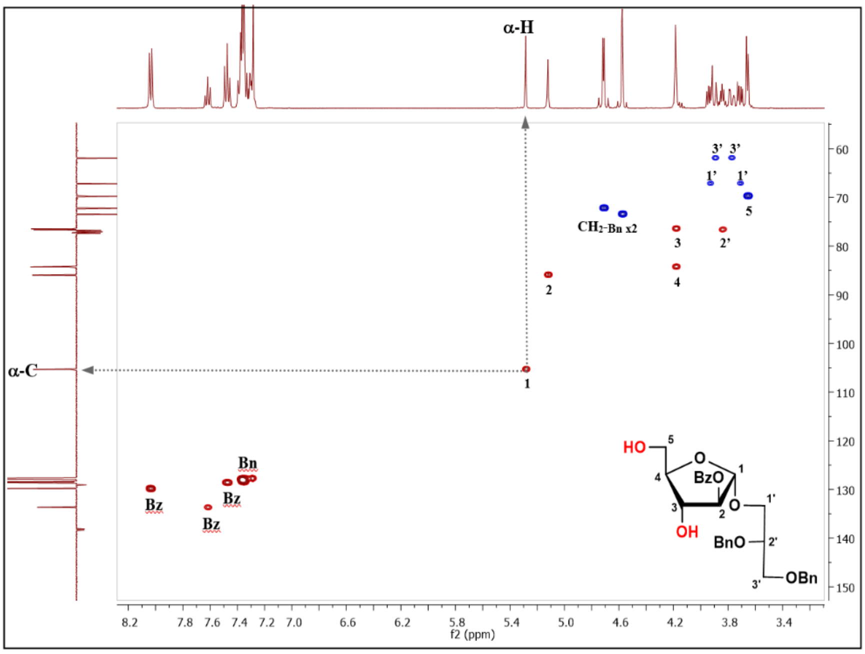

Figure S0: HSQC-NMR spectrum for TAG's acceptor **(5)**.

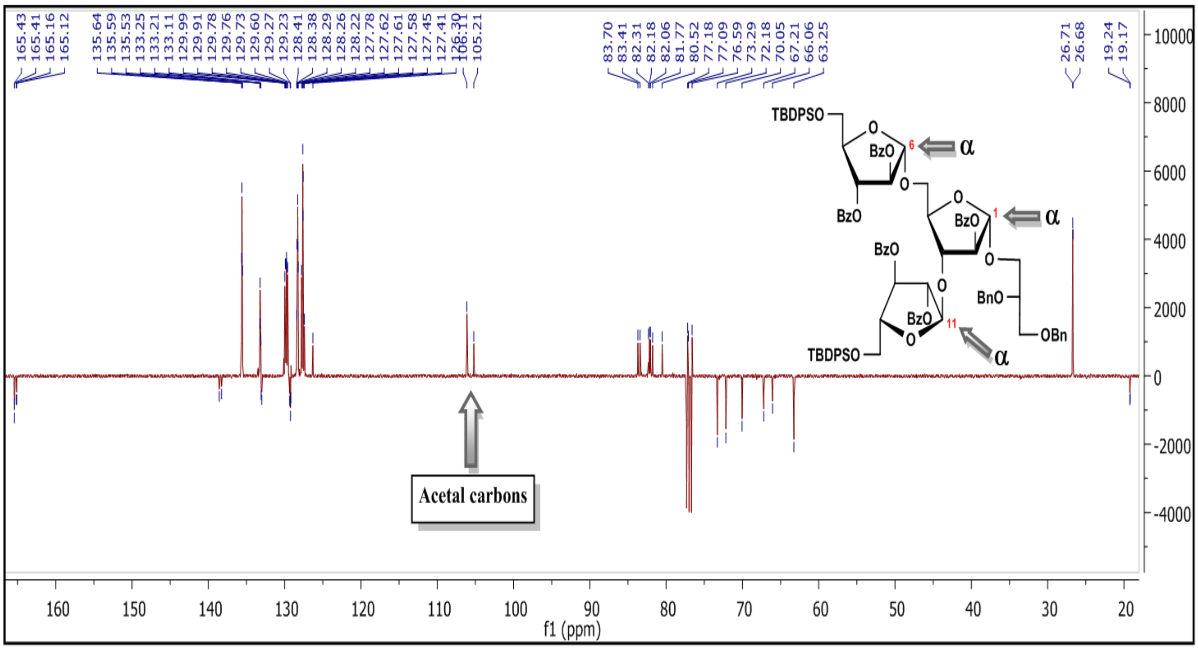

Figure S1: ^{13}C-NMR spectrum for fully protected tri-arabino glycerol **(6)**.

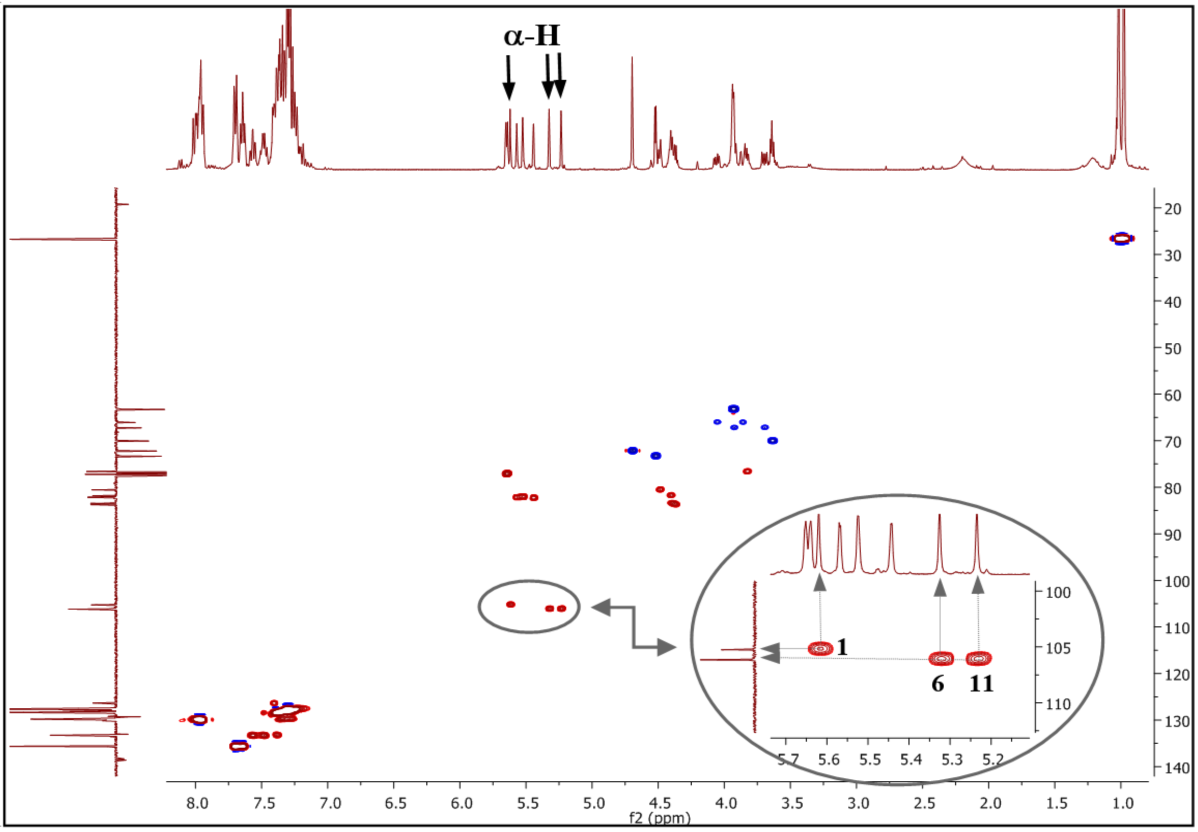

Figure S2: HSQC-NMR spectrum for tri-arabino glycerol **(6)**.

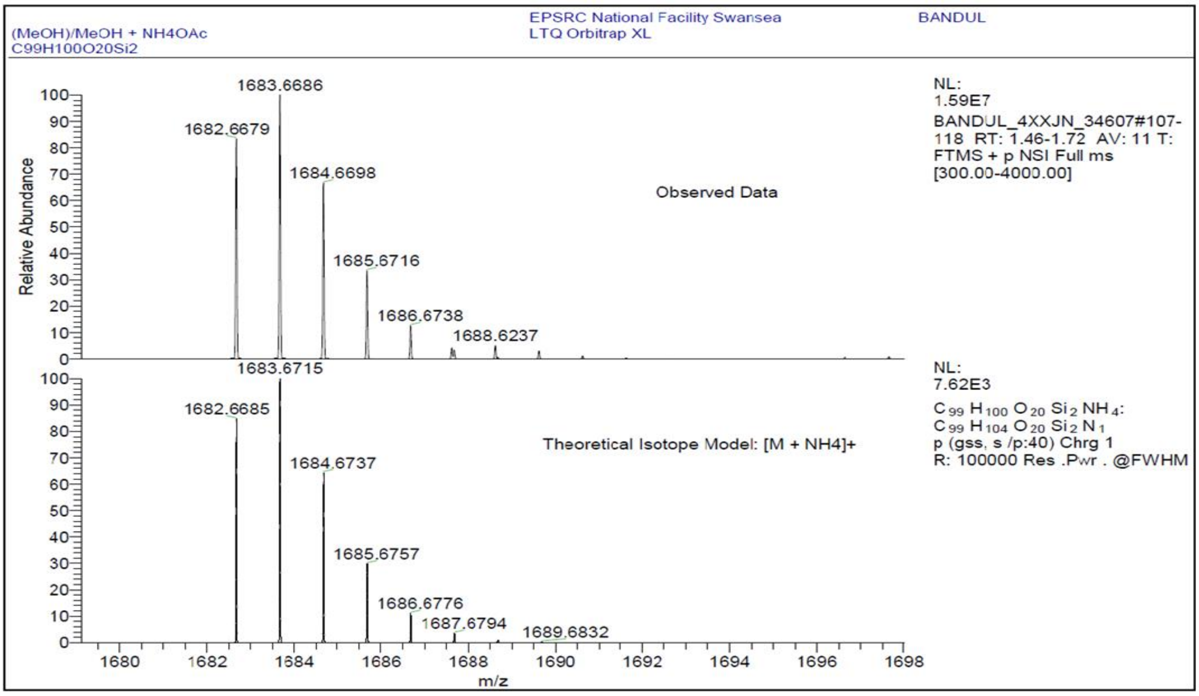

Figure S3: Mass spectrum of the tri-saccharide compound **(6)**.

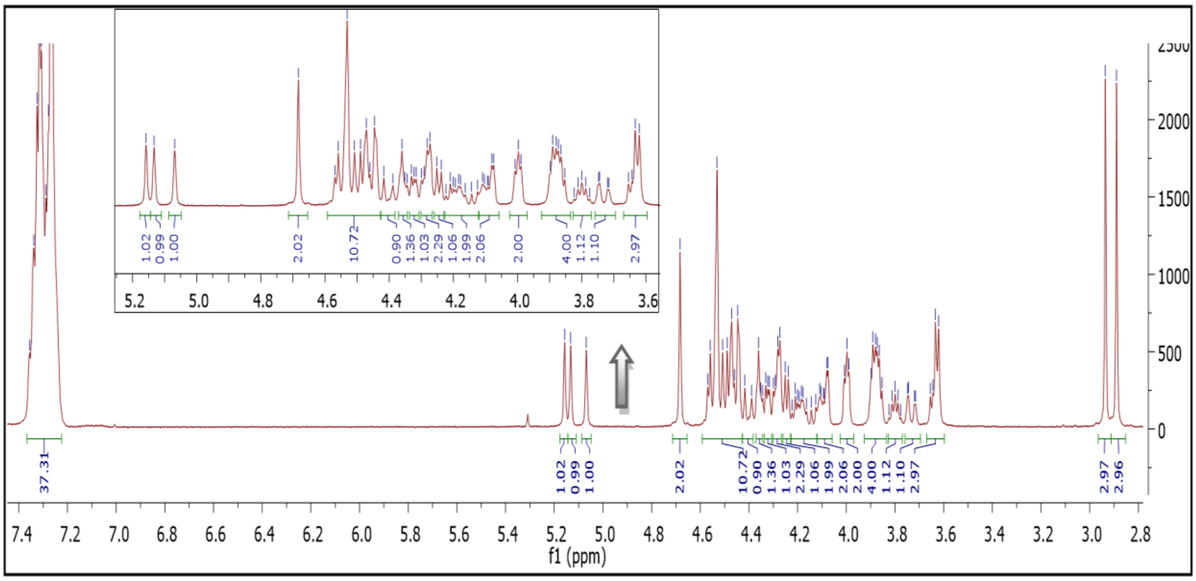

Figure S4: ^{1}H-NMR spectrum for compound **(10)**.

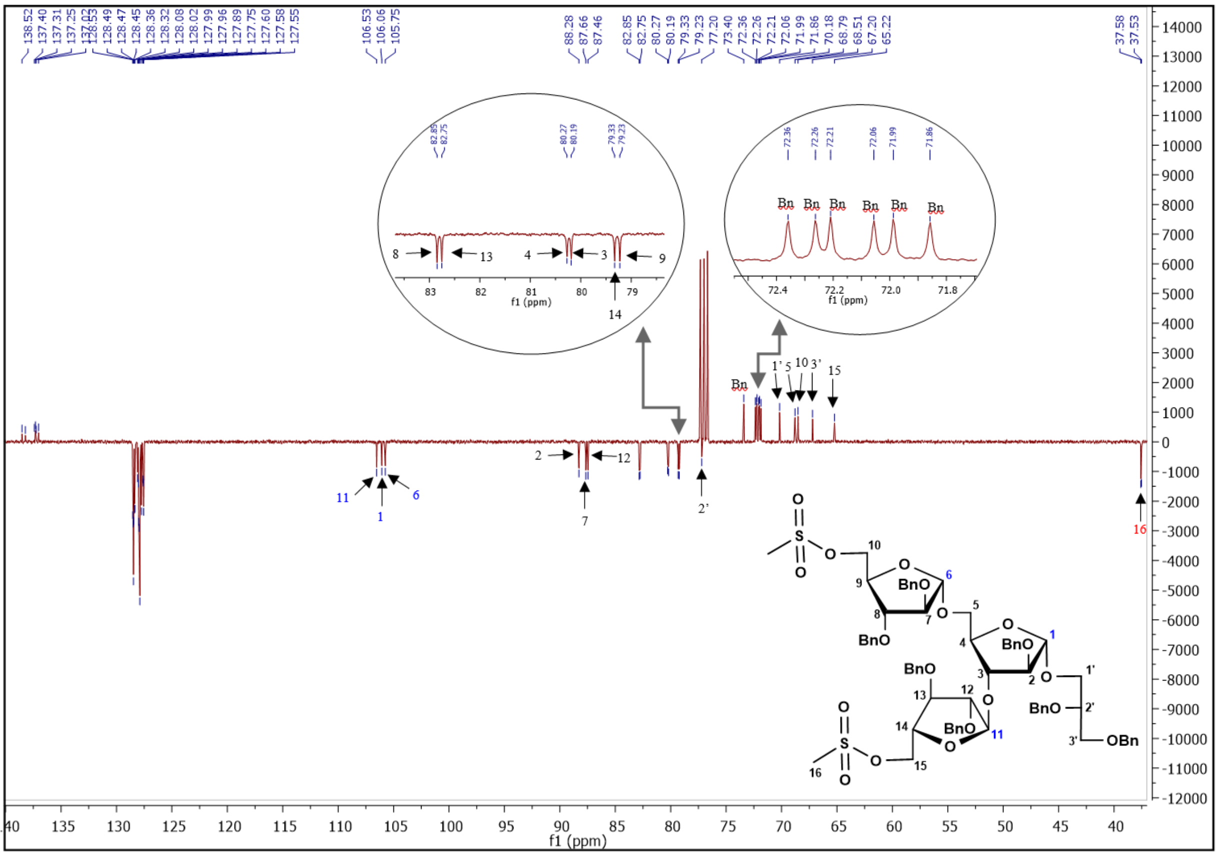

Figure S5: ^{13}C-NMR spectrum for compound **(10)**.

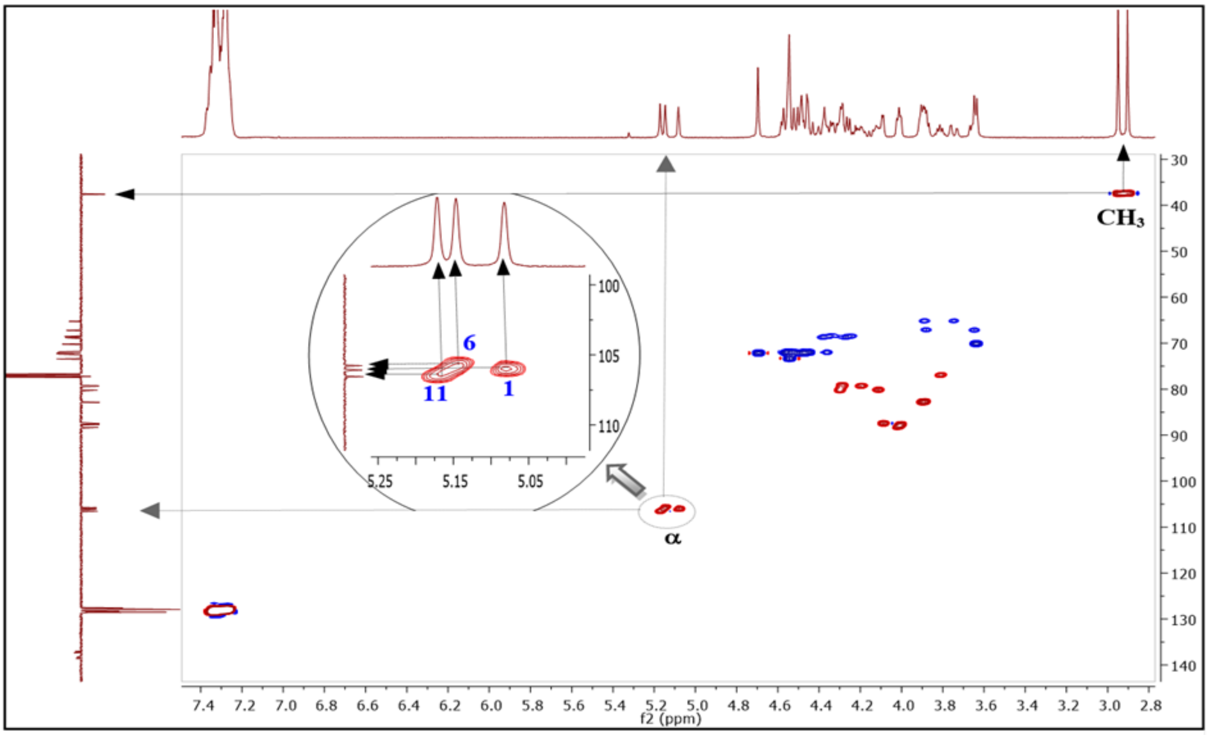

Figure S6: 2D-NMR spectrum for compound (**10**).

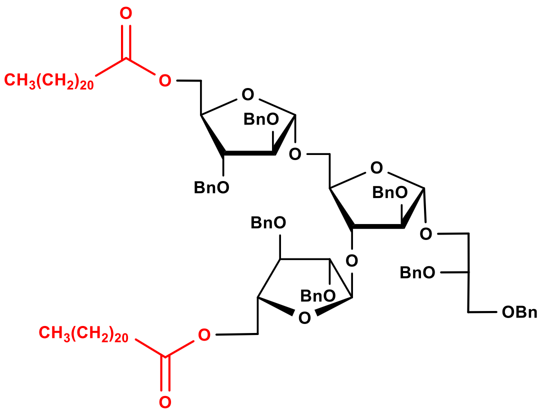

CH3(CH2)20
O
BnO
BnO
BnO
BnO
BnO
OBn
CH3(CH2)20

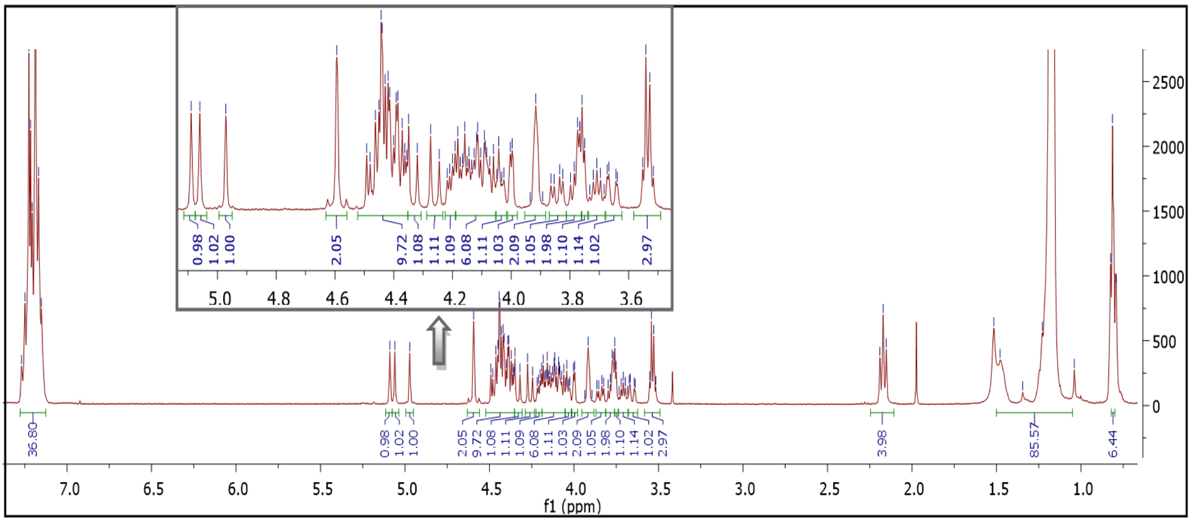

f1 (ppm)

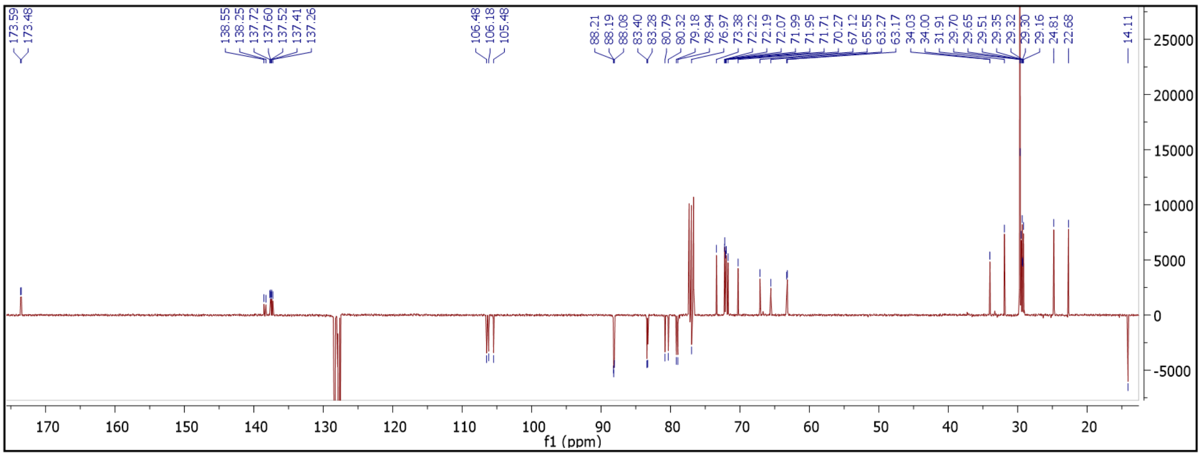

f1 (ppm)

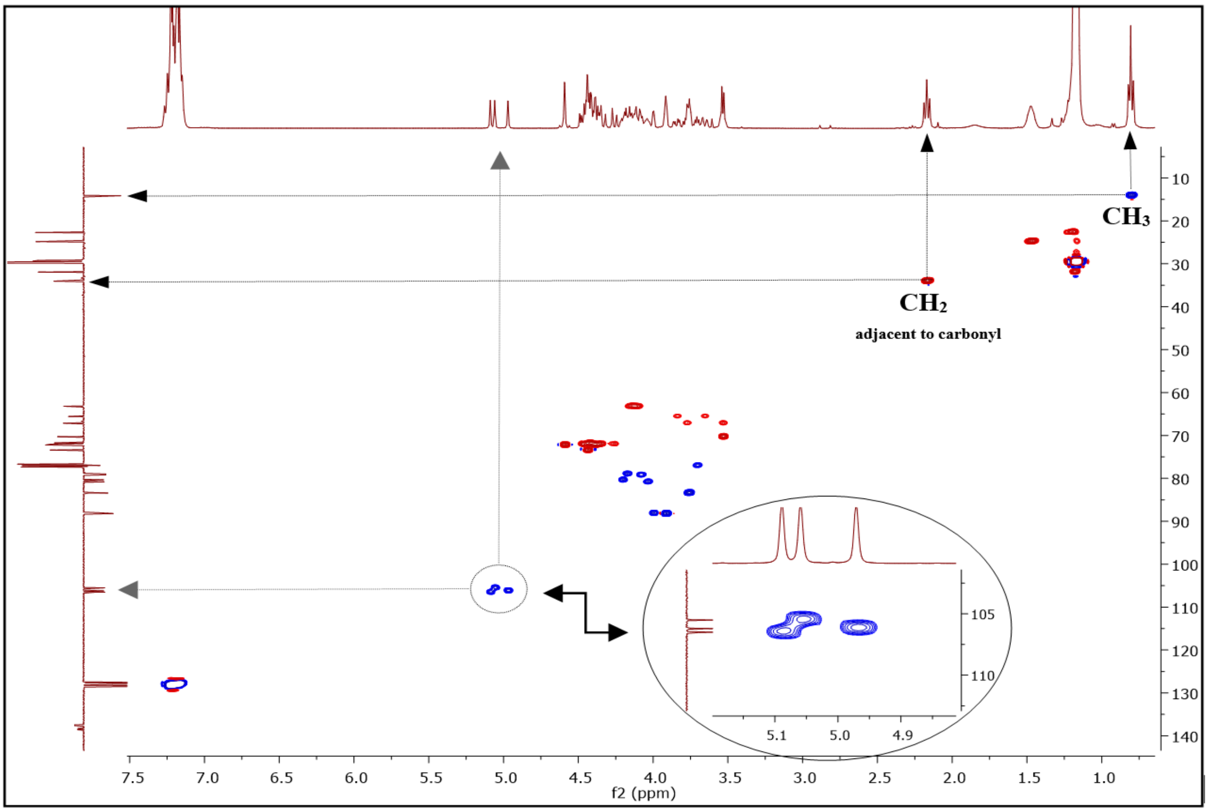

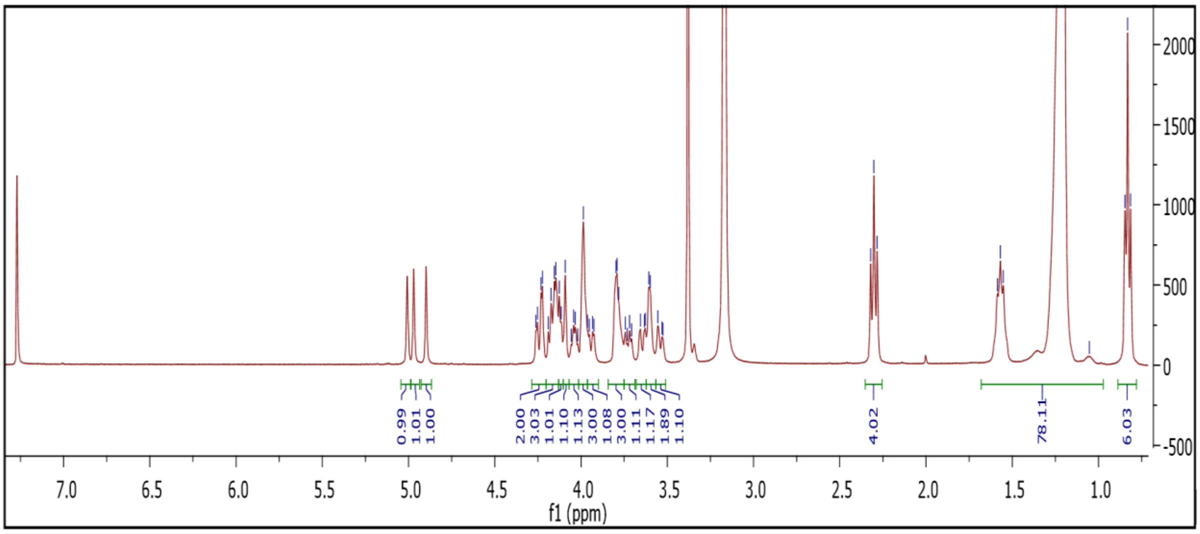

Figure S7: ^{1}H-NMR spectrum for compound **(11,** R = H**)**.

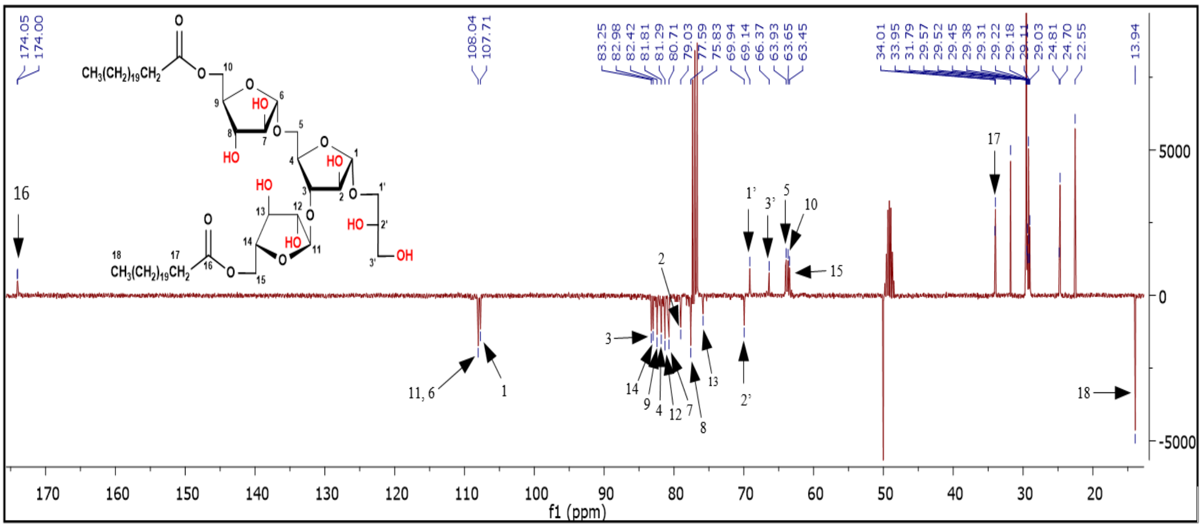

Figure S8: ^{13}C-NMR spectrum for compound **(11,** R = H**)**.

Table S1: The 1H and 13C-NMR data analysis of the glycolipid compound (**11,** R = H).

Proton	Shift	H's	Class	*J/Hz*	Carbon	*δ/ppm*
H_{11}	5.01	1	br. s	-	C_{16}	174.05
H_{1}	4.90	1	br. s	-	C_{1}	107.7
$H_{10, 3}$	4.24	2	br.dd	3.2, 11.7	C_{3}	83.3
$H_{15, 1'', 13}$	4.17	3	br.dd	5.0, 11.8	C_{14}	83.0
$H_{15`}$	4.12	1	br.d	4.3	C_{9}	82.4
$H_{10`}$	4.09	1	m	-	C_{4}	81.8
H_{4}	4.04	1	br.q	5.5	C_{12}	81.3
$H_{2, 7, 9}$	3.95	3	br.m	-	C_{7}	80.7
$H_{5`}$	3.94	1	dd	3.6, 11.5	C_{2}	79.0
$H_{5, 2', 8}$	3.79	3	m	-	C_{8}	77.6
$H_{1'}$	3.73	1	br.dd	4.8, 10.1	C_{13}	75.8
$H_{3''}$	3.64	1	m	-	$C_{2'}$	69.9
$H_{3', 12}$	3.60	2	br.d	3.1	$C_{1'}$	69.1
H_{14}	3.54	1	m	-	$C_{3'}$	66.4
$CH_{2\text{-Next to carbonyl}}$	2.30	4	t	7.6	C_{5}	63.9
$CH_{2\text{-Chain}}$	1.39	83	m	-	C_{10}	63.7
$CH_{3\text{-Terminal}}$	0.83	6	t	6.5	C_{15}	63.5
-	-	-	-	-	C_{17}	34.0
-	-	-	-	-	C_{18}	13.9

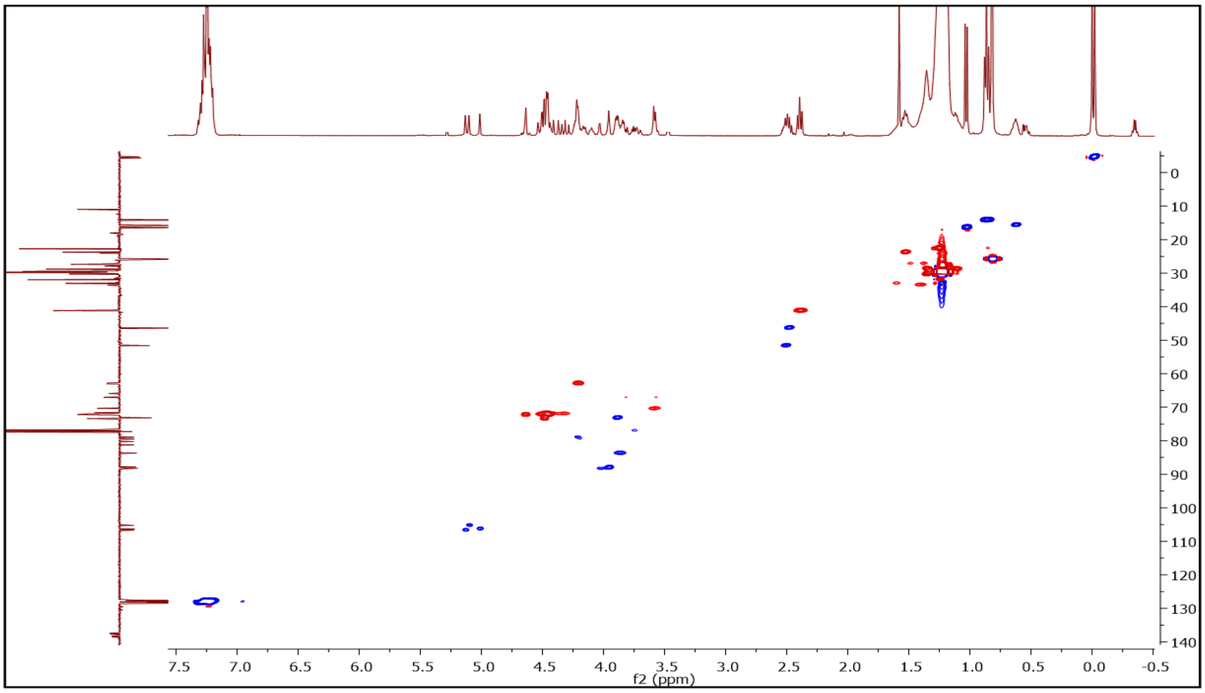

Figure S9: HSQC-NMR spectrum for compound **(13a (R` = TBDMS))**.

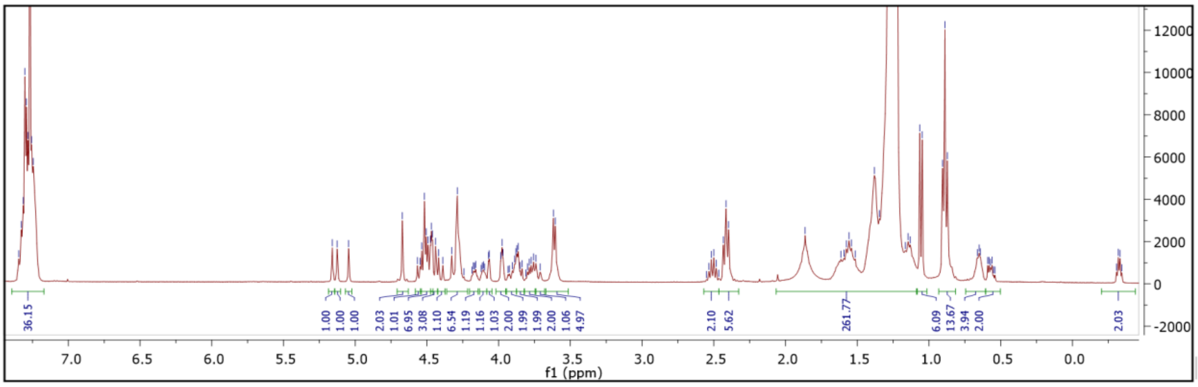

Figure S10: ^{1}H-NMR spectrum for compound **(14a (R` = H)**.

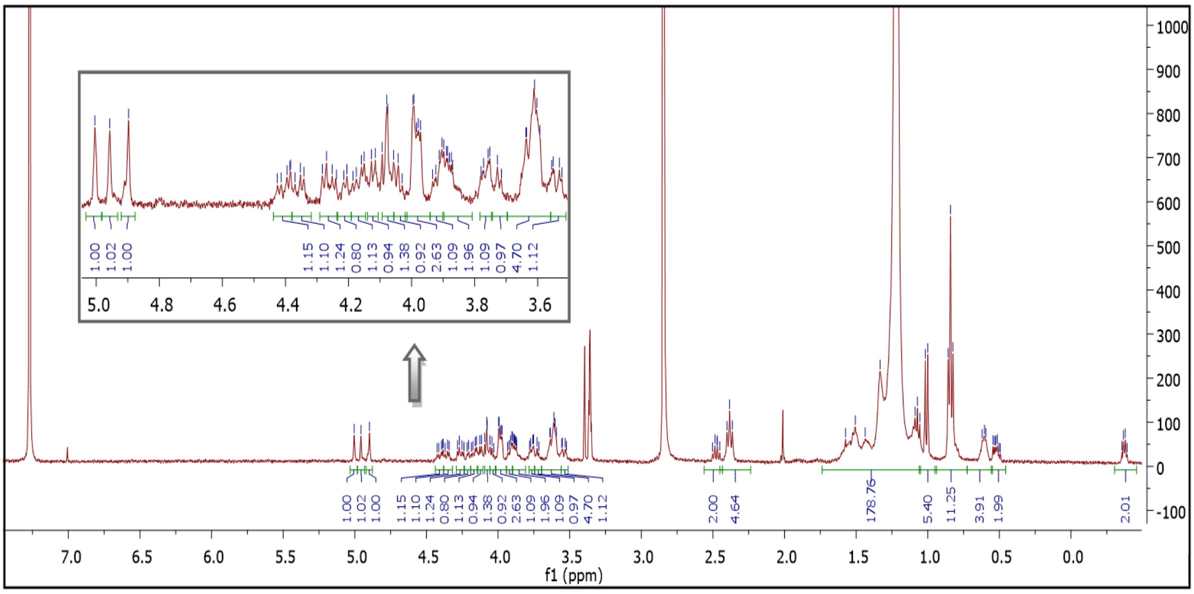

Figure S11: ^{1}H-NMR spectrum for compound **(15a (R` = H))**.

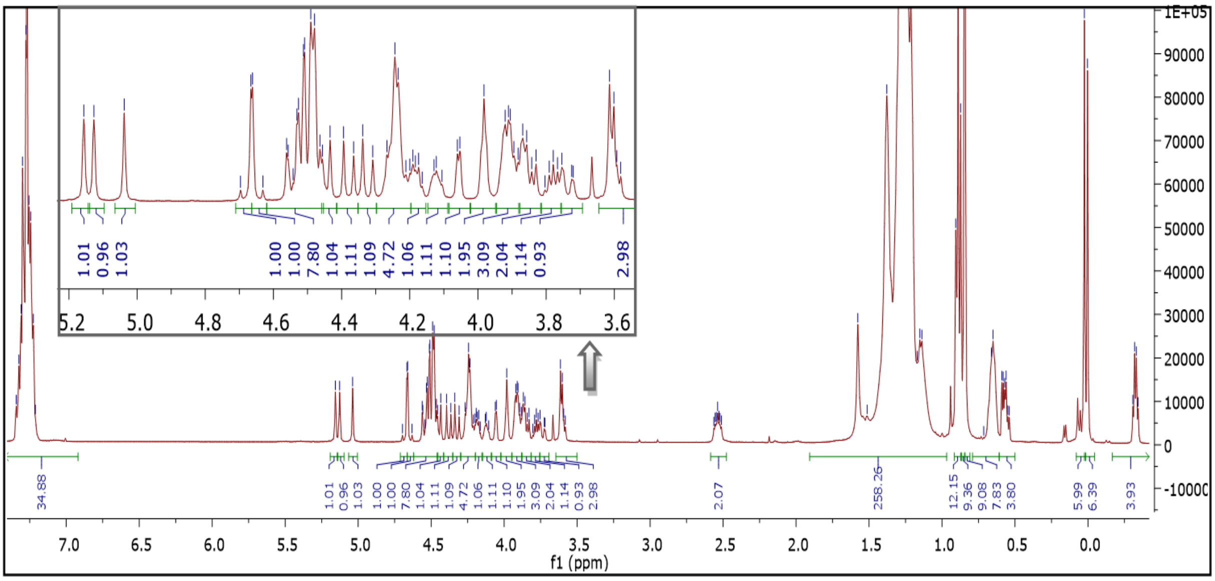

Figure S12: ^{1}H-NMR spectrum for compound **(13b (R` = TBDMS))**.

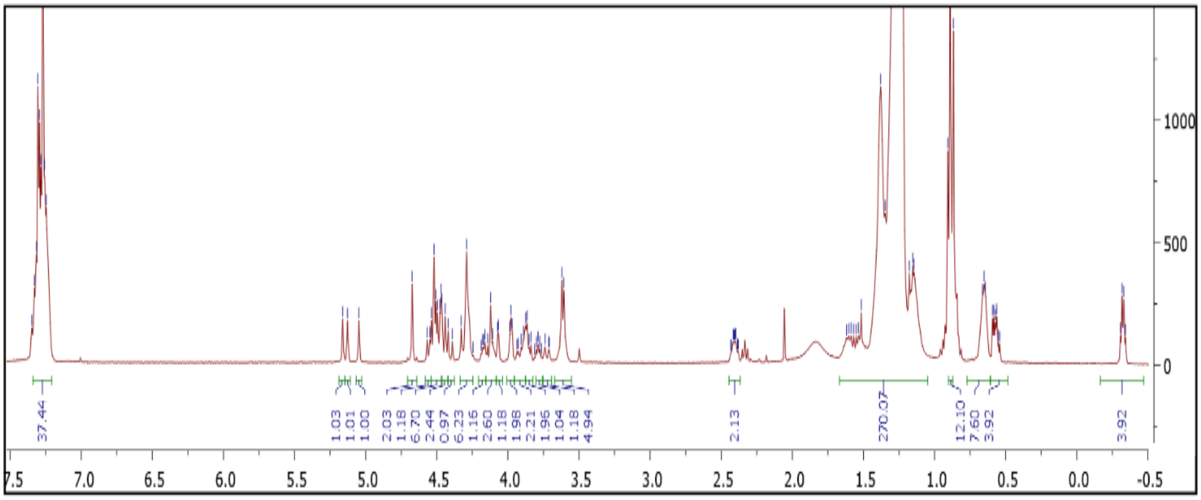

Figure S13: ^{1}H-NMR spectrum for compound **(14b (R` = H))**.

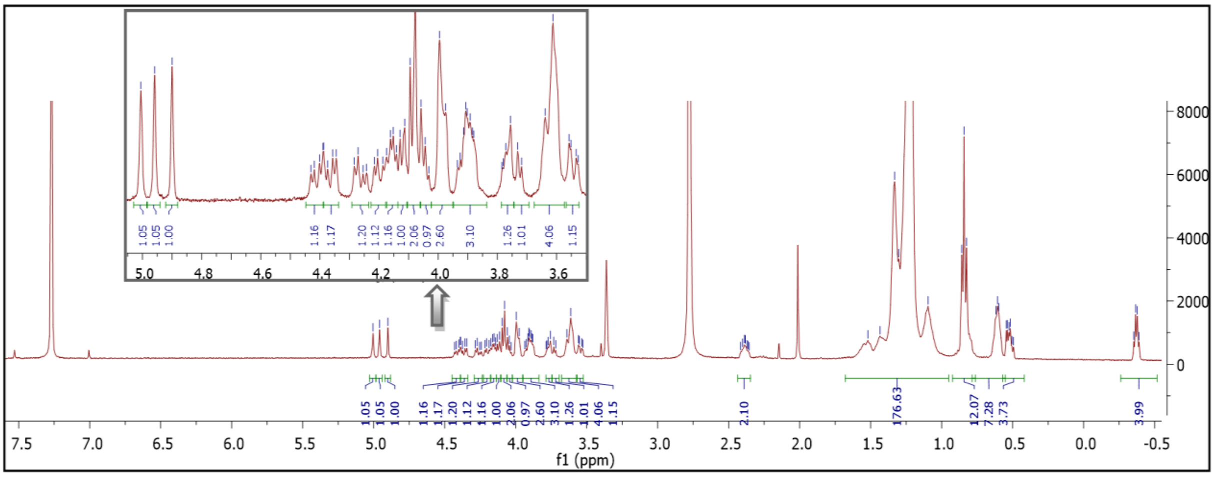

Figure S14: ^{1}H-NMR spectrum for compound **(15b (R` = H))**.

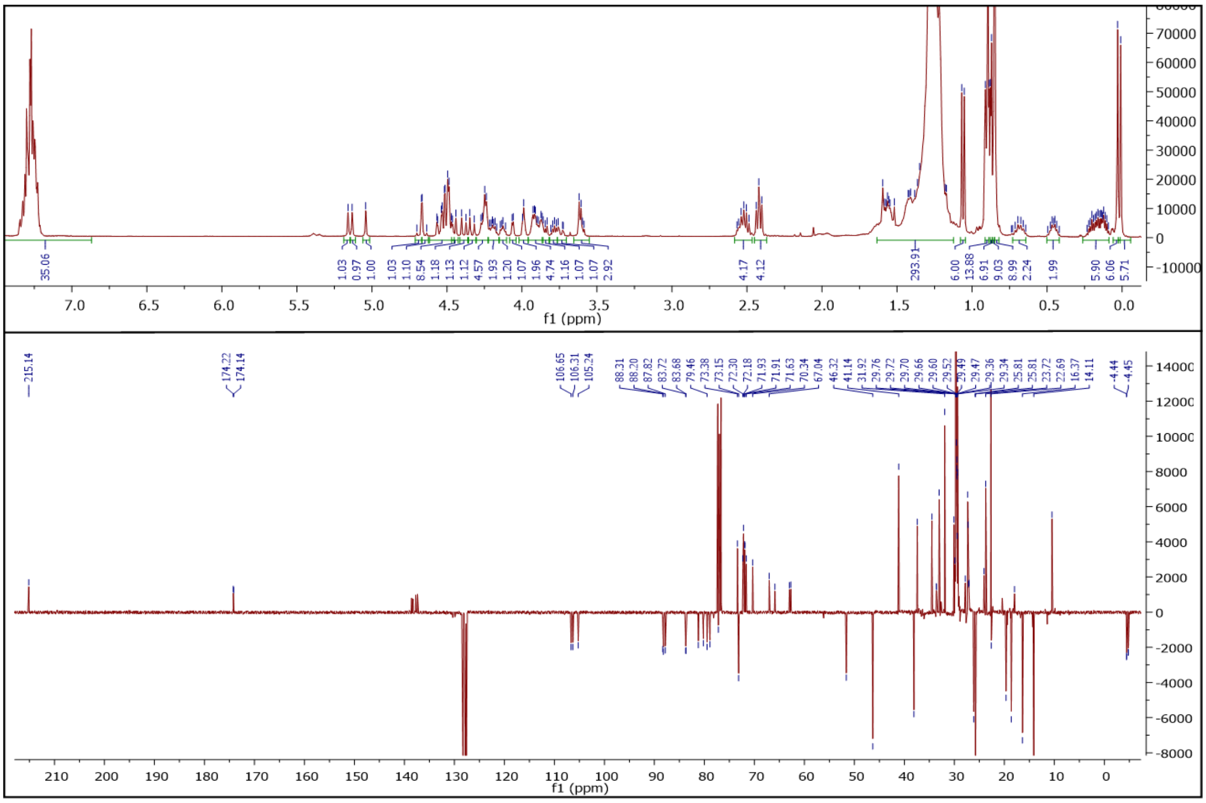

Figure S15: ^{1}H and ^{13}C-NMR spectra for compound **(13c (R` = TBDMS))**.

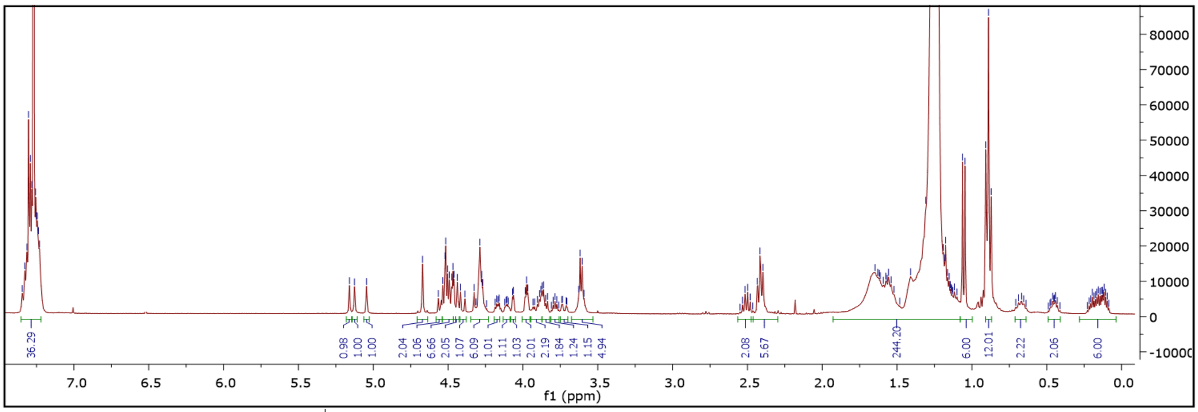

Figure S16: ^{1}H-NMR spectrum for compound **(14c (R` = H))**.

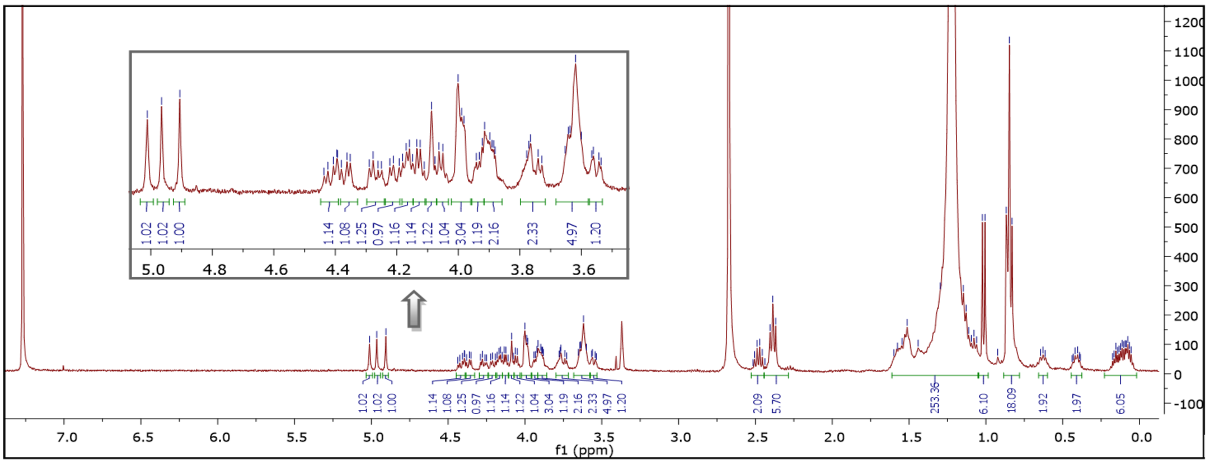

Figure S17: ^{1}H-NMR spectrum for compound **(15c (R` = H))**.
